# Targeted single-cell gene induction by optimizing the dually regulated CRE/*loxP* system by a newly defined heat-shock promoter and the steroid hormone in *Arabidopsis thaliana*

**DOI:** 10.3389/fpls.2023.1171531

**Published:** 2023-06-05

**Authors:** Takumi Tomoi, Toshiaki Tameshige, Eriko Betsuyaku, Saki Hamada, Joe Sakamoto, Naoyuki Uchida, Keiko U. Torii, Kentaro K. Shimizu, Yosuke Tamada, Hiroko Urawa, Kiyotaka Okada, Hiroo Fukuda, Kiyoshi Tatematsu, Yasuhiro Kamei, Shigeyuki Betsuyaku

**Affiliations:** ^1^ Center for Innovation Support, Institute for Social Innovation and Cooperation, Utsunomiya University, Utsunomiya, Japan; ^2^ School of Engineering, Utsunomiya University, Utsunomiya, Japan; ^3^ Laboratory for Biothermology, National Institute for Basic Biology, Okazaki, Japan; ^4^ Kihara Institute for Biological Research (KIBR), Yokohama City University, Yokohama, Japan; ^5^ Division of Biological Sciences, Graduate School of Science and Technology, Nara Institute of Science and Technology, Ikoma, Japan; ^6^ Department of Life Science, Faculty of Agriculture, Ryukoku University, Otsu, Japan; ^7^ Department of Biological Sciences, Graduate School of Science, The University of Tokyo, Tokyo, Japan; ^8^ Biophotonics Research Group, Exploratory Research Center on Life and Living Systems (ExCELLS), Okazaki, Japan; ^9^ Center for Gene Research, Nagoya University, Nagoya, Japan; ^10^ Institute of Transformative Bio-Molecules (WPI-ITbM), Nagoya University, Nagoya, Japan; ^11^ Department of Molecular Biosciences and Howard Hughes Medical Institute, The University of Texas at Austin, Austin, TX, United States; ^12^ Department of Evolutionary Biology and Environmental Studies, University of Zurich, Zürich, Switzerland; ^13^ Center for Optical Research and Education (CORE), Utsunomiya University, Utsunomiya, Japan; ^14^ Robotics, Engineering and Agriculture-Technology Laboratory (REAL), Utsunomiya University, Utsunomiya, Japan; ^15^ Faculty of Education, Gifu Shotoku Gakuen University, Gifu, Japan; ^16^ Laboratory of Plant Organ Development, National Institute for Basic Biology, Okazaki, Japan; ^17^ Ryukoku Extention Center Shiga, Ryukoku University, Otsu, Japan; ^18^ Department of Bioscience and Biotechnology, Faculty of Bioenvironmental Sciences, Kyoto University of Advanced Science, Kyoto, Japan; ^19^ The Graduate University for Advanced Studies (SOKENDAI), Okazaki, Japan; ^20^ Optics and Imaging Facility, Trans-Scale Biology Center, National Institute for Basic Biology, Okazaki, Japan

**Keywords:** *Arabidopsis thaliana*, targeted gene-expression, heat shock response, infrared laser, single-cell analysis, optogenetics, dexamethasone

## Abstract

Multicellular organisms rely on intercellular communication systems to organize their cellular functions. In studies focusing on intercellular communication, the key experimental techniques include the generation of chimeric tissue using transgenic DNA recombination systems represented by the CRE/*loxP* system. If an experimental system enables the induction of chimeras at highly targeted cell(s), it will facilitate the reproducibility and precision of experiments. However, multiple technical limitations have made this challenging. The stochastic nature of DNA recombination events, especially, hampers reproducible generation of intended chimeric patterns. Infrared laser-evoked gene operator (IR-LEGO), a microscopic system that irradiates targeted cells using an IR laser, can induce heat shock-mediated expression of transgenes, for example, *CRE* recombinase gene, in the cells. In this study, we developed a method that induces CRE/*loxP* recombination in the target cell(s) of plant roots and leaves in a highly specific manner. We combined IR-LEGO, an improved heat-shock-specific promoter, and dexamethasone-dependent regulation of CRE. The optimal IR-laser power and irradiation duration were estimated *via* exhaustive irradiation trials and subsequent statistical modeling. Under optimized conditions, CRE/*loxP* recombination was efficiently induced without cellular damage. We also found that the induction efficiency varied among tissue types and cellular sizes. The developed method offers an experimental system to generate a precisely designed chimeric tissue, and thus, will be useful for analyzing intercellular communication at high resolution in roots and leaves.

## 1 Introduction

Intercellular communication is the key to multicellular life. Development is achieved through the coordinated behavior of individual cells within a body. As sessile organisms, plants have developed complex cell-to-cell communication networks to fine-tune their growth and development in response to various internal and external cues (Van Norman et al., 2011; Yadav et al., 2014; Tabassum and Blilou, 2022). For example, in flowering plants, stem cells located in the shoot apical meristem (SAM) and root apical meristem (RAM) are maintained through communication with the surrounding cells through various signaling molecules, such as phytohormones, small peptides, and proteins (Betsuyaku et al., 2011; Gaillochet and Lohmann, 2015). Furthermore, environmental responses are regulated by intercellular communication between cells that detect external stimuli and other bystander cells. For instance, in addition to the well-known systemic acquired resistance, which is triggered by intertissue communication between the primarily infected tissues and the non-infected tissues, local infection with a specific pathogen elicits a spatially distinct and concentric pattern of two phytohormone active domains, inner salicylic acid and outer jasmonic acid domains, around the initial infection site (Fu and Dong, 2013; Betsuyaku et al., 2018).

Genetic approaches using loss-of-function and gain-of-function mutants have been a standard method to obtain functional and mechanistic insights into intercellular communication in plants. In addition, experimental induction of genetic mosaics or chimeras, where cells of different genotypes are included in a single organism, has also contributed to dissecting experimentally intercellular communication. Before the advent of molecular biology, chimeras were induced *via* grafting, callus co-culture, and random transposon insertion (Frank and Chitwood, 2016). Recent developments in molecular tools have enabled the induction of chimeras of highly designed genotypes using the bacteria-derived DNA recombinase system CRE/*loxP* in plants (Frank and Chitwood, 2016). CRE catalyzes DNA recombination in a region flanked by two *loxP* sites, where the sequence is deleted or inverted (Duyne, 2001). This system enables ectopic expression or knockout of a gene of interest. When CRE activity is regulated spatiotemporally, this system enables visualization of the cell lineage using a fluorescent protein in a limited number of cells. Indeed, the CRE/*loxP*-mediated chimera is a powerful experimental system for analyzing cell-to-cell communication, developmental cell lineage, and lethal gene function (Bouyer et al., 2008; Kawade et al., 2010; Tameshige et al., 2013; Zeng et al., 2020). In these studies, spatiotemporal restriction of recombination events was implemented using tissue-specific promoters, other conditional promoters, and chemical-dependent activation of the CRE recombinase. Restricted recombination in cell (s) at a specific time can be further secured by the combinatorial use of a promoter of *HEAT SHOCK PROTEIN* (*HSP*) and a dexamethasone (DEX)-inducible system (Nishihama et al., 2016). Owing to the diffusive nature of heat and drugs, these strategies can be easily applied to induce CRE-mediated recombination in a relatively broader area, but not in single-cell resolution. Moderate heat shock stimuli result in the induction of CRE-mediated recombination in a mosaic fashion, allowing for contingent single-cell recombination due to the stochastic nature of CRE-mediated recombination (Heidstra et al., 2004; Zeng et al., 2020). Although this random single-cell expression may be sufficient for the microscopic study of relatively narrow regions of tissues, such as RAM, identification of such rare events in much broader tissues, such as leaves, becomes laborious. In addition, trade-offs may occur between high inducibility in the targeted cells and leaky expression in unintended cells. These technical difficulties limit their use for various experimental purposes. Therefore, conventional strategies are still widely used to analyze preferable chimeric patterns that are incidentally acquired through the random induction of CRE-mediated recombination.

Thus, the design of versatile and simple methods to induce CRE/*loxP* recombination in a targeted single cell or few cells would facilitate cell-to-cell communication studies. Several protocols have been developed that utilize laser irradiation to warm up specifically targeted single cells in a whole living body, thereby enabling targeted single-cell modification of gene expression (Stringham and Candido, 1993; Sato-Maeda et al., 2006; Kamei et al., 2009). Among these, the infrared laser-evoked gene operator (IR-LEGO) system, characterized by high efficiency and reproducibility of gene induction with negligible cellular toxic photodamage due to the use of an IR laser, has been successfully applied to Arabidopsis roots and female gametophytes along with several animal systems (Deguchi et al., 2009; Kamei et al., 2009; Kobayashi et al., 2013; Shimada et al., 2013; Kawasumi-Kita et al., 2015; Hasugata et al., 2018; Hwang et al., 2019). However, there are no versatile protocols for the use of IR-LEGO in plant systems. The heat shock (HS)-independent leaky transactivity of the conventional promoter *HSP18.2* observed in the vascular tissues of Arabidopsis might also interfere with the effective application of IR-LEGO with the HS-mediated CRE/*loxP* system (Takahashi et al., 1992; Deguchi et al., 2009). In animal fields, optogenetics with light-sensitive protein domains has prospered in such targeted gene expression, however, several of these protein domains were derived from plants. This implies that the system possesses a tendency to influence the intrinsic photoreceptor systems in plants, unless the system itself is designed carefully and precisely (Ochoa-Fernandez et al., 2020; Christie and Zurbriggen, 2021). In this context, establishing versatile and efficient protocols for IR-LEGO combined with relevant plant lines harboring a heat-shock-inducible DNA recombination system can offer an alternative optogenetic method for plant studies (Shikata and Denninger, 2022).

In this study, we report an improved setup for IR-LEGO-mediated heat shock application in Arabidopsis. We identified a low-background HS-inducible promoter region in the promoter sequence of Arabidopsis *HSP18.2*. The combination of the promoter with CRE recombinase fused to the glucocorticoid receptor hormone-binding domain (CRE-GR) accomplished tightly regulated gene expression in targeted single cells in Arabidopsis root and leaf tissues. Furthermore, we screened a range of laser-irradiation conditions for the most effective inducibility in Arabidopsis roots using our new IR-LEGO setup. This robust method can be applied to a wide range of biological events involving cell-to-cell communication in Arabidopsis, thereby stimulating and advancing the study of intercellular communication.

## 2 Materials and methods

### 2.1 Plant materials and growth conditions

*Arabidopsis thaliana* of the wild type (WT) used in this study was Columbia-0 (Col-0), and all other transgenic lines were of the Col-0 background. The plants were grown either under a 16-h light/8-h dark cycle with white light at approximately 50 µmol m^−2^ s^−1^ or under approximately 50–80 µmol m^−2^ s^−1^ of continuous light condition at 22–23°C unless otherwise stated. Soil-grown ones were watered daily with a 0.5 g/L Hyponex solution (Hyponex, Japan).

### 2.2 Construction of transgenic plants

The promoter sequence of the *HSP18.2* gene (p*HSP18.2v2*) was amplified from the Arabidopsis WT genomic DNA using Phusion High-Fidelity DNA Polymerase (New England BioLabs, MA, USA) and cloned into the pENTR/D-TOPO vector (Invitrogen, Thermo Fisher Scientific, MA, USA) according to the manufacturer’s instructions. The p*4xHSEs* sequence containing four tandemly repeated heat shock elements (HSEs) and the minimal sequence of Cauliflower Mosaic Virus *35S* promoter (minimal p*35S*) flanked by AscI and XhoI sites (5′-GGCGCGCCAGAACGTTCTAGAACACTAGAACGTTCTAGAACACTAGTAGAACGTTCTAGAACACTAGAACGTTCTAGAACACTAGTGCAAGACCCTTCCTCTATATAAGGAAGTTCATTTCATTTGGAGAGGACCTCGAG-3′) was chemically synthesized (Eurofins, Luxembourg). Both promoter sequences were digested using AscI and XhoI and cloned into the AscI/XhoI-digested pAM-PAT-GW vector (a gift from Dr. Jane Parker) to generate pAM-PAT-pHSP18.2v2-GW and pAM-PAT-p4xHSEs-GW, respectively. The *GUS* sequence from the pENTR-GUS (Invitrogen, Thermo Fisher Scientific) vector was recombined into these vectors using Gateway™ LR Clonase™ II Enzyme (Invitrogen, Thermo Fisher Scientific). The resulting pAM-PAT-pHSP18.2v2-GUS and pAM-PAT-p4xHSEs-GUS vectors were introduced into *Agrobacterium tumefaciens* GV3101::pMP90RK and then into the Arabidopsis WT plants using the floral dip method (Clough and Bent, 1998). Three (#1, #2, and #3) pAM-PAT-pHSP18.2v2-GUS and five (#1, #6, #7, #17, and #18) pAM-PAT-p4xHSEs-GUS homozygous lines were selected *via* segregation analysis in subsequent generations and analyzed in this study.

The CRE-GR fragment in a pBluescript II SK+-based plasmid (Tameshige et al., 2013) was PCR-amplified and cloned into the pENTR/D-TOPO vector (Invitrogen, Thermo Fisher Scientific) and then transferred to the pAM-PAT-pHSP18.2v2-GW vector using Gateway™ LR Clonase™ II Enzyme (Invitrogen, Thermo Fisher Scientific). From the resulting pAM-PAT-pHSP18.2v2-CRE-GR vector, the p*HSP18.2v2:CRE-GR* sequence was PCR-amplified using Phusion High-Fidelity DNA Polymerase (New England BioLabs), cloned into the pENTR/D-TOPO vector (Invitrogen, Thermo Fisher Scientific), and recombined into the pGWB407 vector (Nakagawa et al., 2007) using Gateway™ LR Clonase™ II Enzyme (Invitrogen, Thermo Fisher Scientific), to finally generate pGWB407-pHSP18.2v2-CRE-GR. The binary vector, through *A. tumefaciens* GV3101::pMP90, was used to transform *A. thaliana* Col-0 wild-type plants *via* floral dip (Clough and Bent, 1998). To minimize possible transgene silencing events, transgenic plants with low-copy T-DNA insertion numbers were selected from 17 isolated independent Kanamycin-resistant T_1_ plants as follows. Genomic DNA was extracted using the DNeasy Plant Mini Kit (QIAGEN), and qPCR was performed to estimate the relative T-DNA copy numbers. The primer sets used are listed in **Supplementary Table S1**. The *ΔCt* values between the GR transgene amplicon and native gene (AT1G71866) amplicon showed some distribution peaks. The plants with the smallest *ΔCt* values were selected as low-copy transformants. After crossing the *loxP* reporter line (p*35S:loxP-GUS-Ter-loxP-VENUS*, Anastasiou et al., 2007), the lines were screened using fluorescence stereomicroscopy. For further experiments, we selected the two best lines, #3 and #10, which showed no yellow fluorescence under normal growth conditions at 22°C and bright yellow fluorescence after HS treatment at 37°C for 45 min on DEX-applied medium, and focused on line #3 for the rest of study.

### 2.3 HS experiment for GUS staining

Selected homozygous lines of pAM-PAT-pHSP18.2v2-GUS and pAM-PAT-p4xHSEs-GUS plants were grown on BM2 soil (Berger, Canada) under the continuous light condition at 23°C. Leaves were sampled from almost 2–3-week-old plants, submerged in distilled water to fully cover the leaf surfaces, and maintained in an incubation chamber (ADVANTEC, Japan) at 37°C or at room temperature (air-conditioned at 23°C) in the dark for 2 h. The treated leaves were then transferred to a GUS staining solution and incubated in the 37°C incubation chamber overnight. For the detailed observation of pAM-PAT-pHSP18.2v2-GUS lines #1-1 and #3-3, the surface-sterilized seeds of the homozygous lines were grown in 1 mL of liquid half-strength Murashige Skoog (1/2 MS) medium supplemented with 1% sucrose in a 24-well chamber for 6 d or grown further on solid 1/2 MS media in a six-well chamber for 5 more days, and then were treated either in the 37°C incubation chamber or at room temperature (air-conditioned at 23°C) under dark conditions for 2 h and reacted in a GUS staining solution (500 µg/ml X-gluc, 200 mM sodium phosphate Buffer pH 7.0, 1 mM potassium ferricyanide, 1 mM potassium ferrocyanide, 2 mM EDTA, 0.2% Triton X-100, 10% methanol) in an incubation chamber overnight at 37°C. The stained seedlings were, then, washed with 70% ethanol. Microscopic images were taken using the DP80 CCD camera with the UPlan FL N 10×/0.30 Ph1 or UPlan FL N 20×/0.50 Ph1 objective lens in the BX53 microscope system controlled by the cellSens software (Olympus, Japan).

### 2.4 HS experiment for evaluation of p*HSP18.2v2*-mediated CRE-GR/*loxP* system

Selected homozygous siblings (#3-3 and #3-7) of the above-mentioned line #3 were grown on solid 1/2 MS media containing 1% sucrose with or without 5 µM DEX under the continuous light condition at 23°C. A part of the non-DEX-treated seedlings were transferred onto solid 1/2 MS media containing 1% sucrose with 5 µM DEX a day before HS experiments. 6-d-old-seedlings were either treated with 23°C for 60 min, 37°C for 10 min, or 37°C for 60 min under a dark condition. All the seedlings were further incubated under the continuous light condition at 23°C overnight, and bright field (BF) and VENUS fluorescence images of the seedlings were captured with the M205FA automated stereomicroscope equipped with DFC365FX camera in 12-bit mode using the AF6000 software (Leica Microsystems, Germany). VENUS images were taken through YFP filter (Leica Microsystems). All BF and VENUS images were captured with the same microscopic settings, respectively, in the same set of experiments (detailed conditions available upon request).

As for the time-course analysis, the selected homozygous lines were grown on solid 1/2 MS media containing 1% sucrose for 5 d under the continuous light condition at 23°C. Then, the seedlings were further grown on new solid 1/2 MS media containing 1% sucrose with 5 µM DEX for 1 d under the same light condition at 23°C, and subjected to HS experiments either at 23°C or 37°C for 60 min. All the seedlings were replaced on new solid 1/2 MS media containing 1% sucrose with 5 µM DEX and subjected to microscopic time-lapse imaging using the automated M205FA stereomicroscope controlled by the AF6000 software (Leica Microsystems) as previously described (Betsuyaku et al., 2018). BF and VENUS images were analyzed using the AF6000 software (Leica Microsystems) and presented.

As for the detailed observation of VENUS expression in roots and leaf tissues upon whole-tissue-HS treatment in the presence of DEX, the selected homozygous lines were grown on solid 1/2 MS media containing 1% sucrose for 7 d under the continuous light condition at 23°C. Then, the seedlings were further grown on solid 1/2 MS media containing 1% sucrose with 5 µM DEX for 1 d, and under the same light condition at 23°C. The leaf and root images were taken by TCS SP8 X confocal microscopy equipped with a white light laser at 70% power using the objective lens HC FLUOTAR L 25x/0.95 W VISIR (Leica microsystems). VENUS (500–550 nm, time-gated within 2–12 ns, detected by a HyD detector), chlorophyll autofluorescence (600–700 nm, detected by a normal PMT detector) and transmission images were acquired using 488 nm at 10% laser power.

### 2.5 Local gene induction with the IR-LEGO system

For sample preparation, 5–6 surface-sterilized seeds were placed on the dividing line between the glass surface of the 35-mm glass bottom dishes (Corning, NY, USA) and the cut surface of 1/2 MS medium containing 5 g/L MES-KOH (pH 5.8), 3 mg/L thiamine hydrochloride, 5 mg/L nicotinic acid, 0.3 mg/mL pyridoxine hydrochloride, 1% (w/v) sucrose, and 5 μM DEX, and solidified with 0.5% (w/v) gellan gum (**Supplementary Figure S3**). The seeds were stratified at 4°C for 3 d in the dark and cultured at 22°C for 3 d under approximately 50 µmol m^-2^ s^-1^ of continuous white light from fluorescent lamps.

Using lines #3-3 and #3-7, heat-inducible gene induction was performed using an IR-LEGO 1000 system (Sigma-koki, Japan) in single root cells at intervals of approximately 1 mm from the root tips. The cells were heated by irradiation with an infrared (1,480 nm) laser for 60 s or 1 s with the indicated laser output through a custom-made objective lens UAPO 40×/0.90 NA (Olympus) on an IX-81 inverted optical microscope (Olympus). BF images were acquired after IR-laser irradiation using a scientific CMOS camera (ORCA-Flash 4.0, Hamamatsu Photonics, Japan). The samples were subsequently replaced and cultured at 22°C for 1 d under continuous white light.

For propidium iodide (PI) staining, the solid medium below the root tips was removed, and 200 and 300 μL of 10 μg/mL PI solution was added to the upper and lower parts of the sample. The samples were incubated for 30 min (**Supplementary Figure S3**) and then washed with the same volume of distilled water for 10 min. The samples were subsequently subjected to confocal microscopy analysis.

Fluorescent signals from VENUS and PI in roots were observed under an IX-71 inverted optical microscope (Olympus) equipped with a CSU-X1 confocal scan head (Yokogawa, Japan). To identify root cells possessing VENUS or PI signals, 50–60 z-stack images of 1,024 × 1,024 pixels were acquired at 2-μm steps between optical sections using the objective lens UPlanSApo 20×/0.75 NA, 488-nm and 561-nm laser (60 and 50 mW, respectively), and a scientific CMOS camera (ORCA-Flash 4.0, Hamamatsu Photonics, Japan) with the z-stream mode of Metamorph (Molecular Devices, CA, USA). The emission filter sets U-MNIBA3 510–550 nm (Olympus) and U-FRFP 570-625 nm (Olympus) were used to detect the Venus and PI signals, respectively.

To obtain three-dimensional reconstruction images using the Imaris software (Bitplane, Oxford Instruments, UK), 313 z-stack images of 1,024 × 1,024 pixels were acquired using a confocal microscope NikonA1 (Nikon, Japan) at 0.385-μm steps between optical sections with the objective lens LWD Lambda S 40×WI/1.15 NA, and 488-nm (40 mW, 8.0% output) and 561-nm (10 mW, 10.0% output) laser. The emission filter sets of 500–550 nm and 570–620 nm were used to detect the Venus and PI signals, respectively.

For IR-LEGO experiments using leaf tissues, leaf disks were prepared using biopsy punches (2 mm in diameter, KAI Medical, Japan) from 14-day-old mature leaves fully infiltrated with 1/2 MS liquid media containing 5 µM DEX. Subsequently, the disks were mounted between the cover glass and a piece of 1/2 MS solid medium supplemented with 5 µM DEX within a 35mm glass bottom dish (IWAKI, Japan, see the text). The prepared leaf-disk specimens were subjected to local HS treatment using a custom-made objective lens UPlanSApo 20×/0.75 NA (Olympus) installed to an IR-LEGO 1000 system (Sigma-koki) based on IX-83 inverted optical microscope (Olympus). BF images were acquired after IR laser irradiation using a scientific CCD camera (ORCA-R2, Hamamatsu Photonics). Subsequently, the samples were further cultured at 23°C for 1 d under continuous white light.

Fluorescent signals from VENUS and PI in leaves were observed using TCS SP8 X confocal microscope system equipped with a white light laser at 70% power using the objective lens HC FLUOTAR L 25x/0.95 W VISIR (Leica microsystems). VENUS (525–580 nm, time-gated within 2–12 ns, detected by a HyD detector), chlorophyll autofluorescence (600–716 nm, detected by a normal PMT detector) and transmission images were acquired using 514 nm at 50% laser power.

### 2.6 Statistical modeling

To estimate the maximum likelihood model for CRE/*loxP* recombination probability and IR-laser power, we employed a series of logistic models. The experimental results were formatted into binary data (1 or 0), representing whether recombination was observed. To explain this objective variable from the explanatory variable of the emitted laser power, a logistic regression model was fitted. Cell death data modeling was performed in the same manner.

A logistic model can be formulated as follows.


(1) $$p=\frac{1}{1+exp(-(ax+b))}$$

where *p* is the probability of CRE/*loxP* recombination, *x* is the IR-laser power (mW), and *a* and *b* are the coefficient and intercept, respectively.

Fitting of such a logistic model can be implemented by glm function (package *stats*) in R with a simple script like “glm(y ~ x, data=data, family=binomial(link=“logit”))”. This standard regression approach was applied for most of the data, except for the data of CRE/*loxP* recombination in single target cells irradiated for 60 s. These data appeared to reach the limit of approximately 40% of recombination occurrence in the range of laser powers from 8.5–14 mW. A special logistic regression was performed to model the data with an upper bound on recombination probability. In this case, the following modified logistic model was applied:


(2) $$p=lim\frac{1}{1+exp(-(ax+b))}$$

where *lim* is the upper limit of recombination probability. *p*, *x*, *a*, and *b* are the same as those above. For maximum likelihood estimation of the parameter *lim*, and *a* and *b*, the Markov chain Monte Carlo (MCMC) method implemented in Stan was used. A custom Stan code was run in R using the package *cmdstanr* 0.3.0.9000. The convergence and absence of problems in Markov chains were confirmed by the cmdstan_summary method of the package. Subsequently, we adopted median values from the obtained posterior samples of *lim*, *a* and *b*. Further details are provided in the Github repository (https://github.com/ttameshige/logistic_model_IR-laser_Cre-loxP).

The target cell sizes were manually measured using the Fiji software (ImageJ; https://imagej.net/Fiji) from images after IR laser irradiation under an IX-81 inverted optical microscope. To reveal the effects of cell size on recombination and cell death probabilities, a series of cell size-filtered subset data were analyzed by logistic regression, as described above. Line plots of the logistic models were drawn using R package *ggplot2*.

Statistical tests to determine the effects of cell size on the response to the IR laser were performed using the likelihood-ratio test. In the cases where the glm function was used for logistic regression, the likelihood-ratio test between cell size-included and -ignored models was performed using the lrtest function in R package *lmtest* 0.9-40. The formulae of the models to be compared are (3) and (1),


(3) $$p=\frac{1}{1+exp(-(a_{1}x+a_{2}c+a_{3}xc+b))}$$

where *p* and *x* are the same as those in equation (1), and *c* is the normalized cell size. In Bayesian logistic modeling with MCMC sampling, the model formulae are (4) and (2)


(4) $$p=lim\frac{1+a_{4}c}{1+exp(-(a_{1}x+a_{2}c+a_{3}xc+b))}$$

for cell-size-included and -ignored models, respectively. In the formula, *p*, *x* and *c* are the same as those above. Parameters *a*_1_–*a_4_*, *b*, and *lim* were estimated using the MCMC sampling method. Likelihood-ratio test for Bayesian model by MCMC method was performed with just basic R functions instead of lrtest because the model cannot be input directly into lrtest function above, which requires specific class of object such as ones output from glm function. Briefly, the likelihood was calculated for each of the cell size-included and -ignored models according to the definition that likelihood equals to the product of all probabilities with which the data are observed if the considering model is true. Then, the deviance was obtained by multiplying -2 and the log-transformed likelihood of the model. The difference between the deviances follows a Chi-square distribution under the null hypothesis that the reduced model, i.e. cell size-ignoring model in this study, is true. Thus, the *p*-value is calculated by the pchisq function (R package *base*) as it gives the probability with which a statistic is observed under a Chi-square distribution. The codes are shared in the Github repository (https://github.com/ttameshige/logistic_model_IR-laser_Cre-loxP).

## 3 Results

### 3.1 Identification of a low-background HS promoter, p*HSP18.2v2*

The promoter sequence of *HSP18.2* (p*HSP18.2*) has been widely employed as an HS-inducible promoter, often in combination with the CRE/*loxP* system in plants (Takahashi et al., 1992; Sieburth et al., 1998; Heidstra et al., 2004; Zeng et al., 2020). Although the system has successfully enabled HS-mediated conditional and/or mosaic expression of genes of interest in various aspects of Arabidopsis biology, a GUS reporter-based histochemical analysis indicated HS-independent p*HSP18.2* activity in the vasculature of young seedlings in addition to HS inducibility in whole seedlings (Takahashi et al., 1992; Deguchi et al., 2009). Such leaky activity might lead to complication in data interpretation or cause an issue when genes of interest harbor morphogenic or cytotoxic effects. Indeed, our trial to express pathogen-derived effector genes in the p*HSP18.2*-driven CRE/*loxP* system failed to obtain appropriate transgenic plants for HS analysis, presumably because of the lethality caused by leaky expression of cell death-inducing effector genes (data not shown).

**Figure 1:**
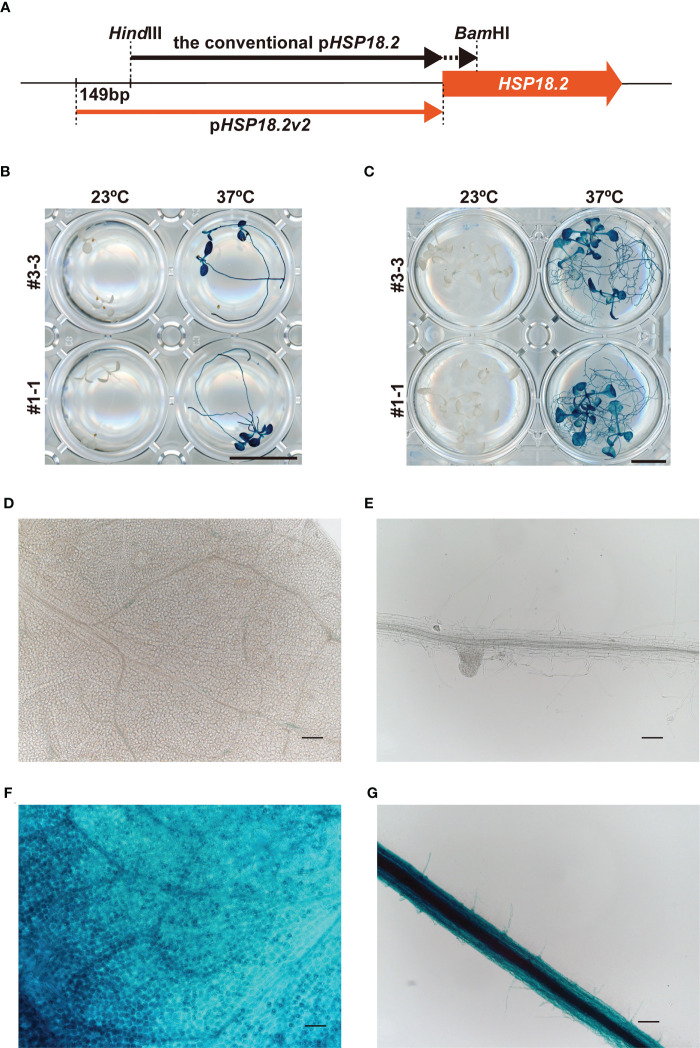
Characterization of the p*HSP18.2v2*. **(A)** Schematic comparison of p*HSP18.2v2* and conventional p*HSP18.2*. The arrows indicate the lengths and positions of both promoters. The thick orange arrow indicates the *HSP18.2* gene. **(B, C)** p*HSP18.2v2*-driven GUS activity after treatment at 23°C or 37°C for 2 h in young **(B)** and mature **(C)** Arabidopsis seedlings. **(D, E)** Representative magnified images of leaf tissue in mature seedlings **(D)** and root tissue in young seedlings **(E)** of line #3-3 after treatment at 23°C. **(F, G)** Representative magnified images of the leaf tissue of mature seedlings **(F)** and the root tissue of young seedlings **(G)** of line #3-3 after treatment at 37°C. Scale bars represent 10 mm in **(B, C)**, and 100 µm in **(D–G)**.

The transactivation of *HSP* genes is regulated by the binding of heat shock factors (HSFs) to HSEs in the promoter regions (Ohama et al., 2017; Bourgine and Guihur, 2021). In animal systems, synthetic promoters consisting of multiple HSE repeats have been successfully shown to induce genes of interest in an HS-specific manner (Bajoghli et al., 2004). Based on this background, we first compared two different designs of HS-inducible promoters, namely, an intrinsic *HSP18.2* promoter (p*HSP18.2*) and a synthetic promoter (p*4xHSE*, see Materials and methods) containing four tandemly repeated HSEs and the minimal p*35S*, with our vector system and plant materials (**Supplementary Figure S1A**). Here, p*HSP18.2* corresponds to the –1 bp to –1,000 bp genomic region of *HSP18.2* (AT5G59720). The GUS coding sequence was fused with these promoters and introduced into Arabidopsis plants of Col-0 accession (**Supplementary Figure S1**). Stable transformants carrying the p*4xHSE : GUS* and p*HSP18.2:GUS* constructs (five and three lines, respectively) were analyzed. In the initial screening of leaves, only four lines (lines #1, #6, #17, and #18) out of the five p*4xHSE* lines exhibited GUS activity with various degrees and patterns upon HS treatment (**Supplementary Figure S1B**). Line #17 exhibited GUS activity without HS treatment. In contrast, all p*HSP18.2:GUS* plants showed clear GUS signals in an HS-dependent manner, suggesting that p*HSP18.2* was an appropriate HS-specific promoter, rather than p*4xHSE* (**Supplementary Figure S1B**). This HS-dependent activity of p*HSP18.2* does not accord with a previous report showing p*HSP18.2* activity in the vasculature without HS treatment (Takahashi et al., 1992; Deguchi et al., 2009). The previous study used the 851 bp promoter sequence, while we cloned a 1,000 bp promoter region upstream of *HSP18.2* (**Figure 1A**). These data indicated that the 149 bp extension to the conventional p*HSP18.2* sequence might contain a regulatory sequence suppressing the vasculature activity of the conventional p*HSP18.2*. This new version of the p*HSP18.2* sequence was designated as p*HSP18.2v2*, which stands for p*HSP18.2 version 2* (**Figure 1A**; **Supplementary Figure S1A**). The following detailed analysis using the homozygous lines of p*HSP18.2v2:GUS* plants demonstrated that p*HSP18.2v2* exhibited high HS-specific activity in the seedlings at two different stages without “leaky” activity in the absence of HS (**Figures 1B, C**). Even after overnight GUS staining reaction at 37°C, one line (#3-3) showed only faint GUS staining around the shoot apex and, very occasionally, along the leaf vasculature, which might indicate weak activity of p*HSP18.2v2* in the tissues of mature plants or reflect the position effect of transgene insertion in the genome of this specific line (**Figures 1C, D**). Adequate HS treatment induced GUS activity in all the examined tissues (**Figures 1B–G**). Notably, there also exists variation in HS sensitivity in different cell types. For example, stomatal guard cells and vasculature showed strong GUS signals (**Figures 1F, G**). Nevertheless, these data show that p*HSP18.2v2* can serve as a tightly regulated HS-inducible promoter in all cell types in Arabidopsis seedlings, in contrast with our initial idea to establish an HS-specific promoter with a synthetic HSE-containing sequence.

### 3.2 p*HSP18.2*v2 with GR to enhance the HS-dependency of CRE/*loxP* system

**Figure 2:**
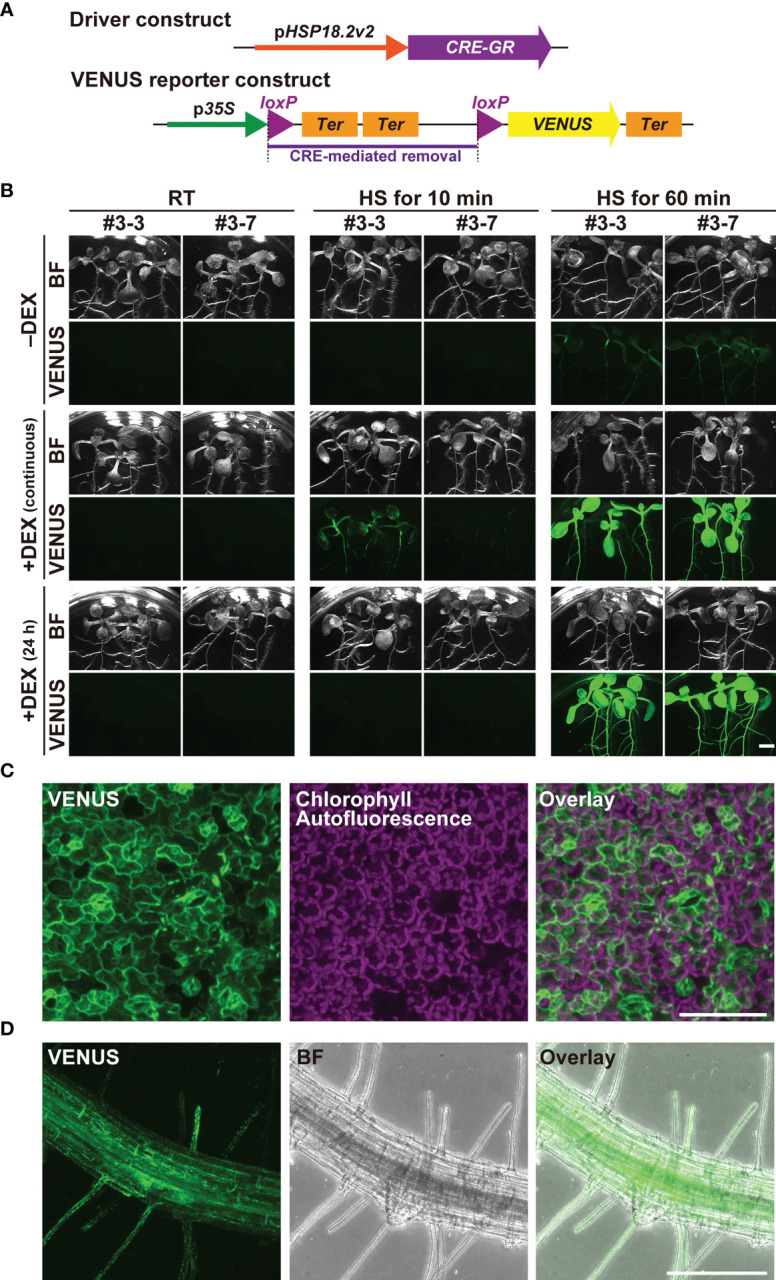
Characterization of the p*HSP18.2v2*-mediated CRE-GR/*loxP* system in Arabidopsis. **(A)** Schematic drawing of the driver- and reporter-constructs used in this study. In the driver construct, the *CRE-GR* fusion gene is driven by p*HSP18.2v2*. In the VENUS reporter construct, p*35S* and an endoplasmic reticulum-resident form of the *VENUS* gene followed by a transcriptional terminator were disrupted by a sequence containing double transcriptional terminators flanked by two *loxP* sites. In the presence of CRE recombinase activity, the region flanked by two *loxP* sites, shown by a purple bar, was removed, and then, *VENUS* was expressed. **(B)** Images of transgenic seedlings containing both the driver- and reporter-constructs after various treatments. Three seedlings of two lines grown either on normal 1/2MS media (-DEX) or on 1/2MS media containing DEX (continuous) were treated either at 23°C (room temperature; RT) for 60 min, at 37°C (HS) for 10 min, or at 37°C (HS) for 60 min. A set of seedlings was transferred onto 1/2MS media containing DEX a day before the temperature shift experiment (24 h) and was subjected to the same treatment. All the samples were further incubated at 23°C under the continuous light overnight, and then photographed using the fluorescent stereomicroscope. This experiment was repeated two times with a similar result. Bright field (BF) and VENUS images from a representative set of experiments are shown. **(C)** Maximal intensity projection of the magnified confocal images of the leaf tissue of line #3-7 **(C, D)** and root tissue of line #3-3 **(D)** of the seedlings treated at 37°C for 60 min. The images were taken after overnight incubation as in **(B)**. VENUS, chlorophyll-derived autofluorescence, and the merged images **(C)**, and VENUS, BF, and the merged images **(D)** are shown. Scale bars represent 2.5 mm **(B)**, 75 µm, **(C)** and 250 µm **(D)**.

The finding that p*HSP18.2v2* is a low-background and versatile HS-inducible promoter prompted us to examine its utility in the CRE/*loxP* system. To minimize unexpected leaky transgene expression by p*HSP18.2v2* during the entire lifetime of Arabidopsis, we generated transgenic Arabidopsis plants carrying a gene to express CRE-GR under the control of p*HSP18.2v2* (p*HSP18.2*v2*:CRE-GR*), thus, enabling dual regulation of CRE activity by DEX and HS (**Figure 2A**). As a reporter, we used a previously reported transgenic line carrying p*35S:loxP-2xTer-loxP-VENUS*, in which the p*35S* and *VENUS* sequences were disrupted by double transcriptional terminators flanked by two *loxP* sites (Anastasiou et al., 2007; Tameshige et al., 2013). These reporter plants showed VENUS fluorescence in the endoplasmic reticulum only after the induction of CRE activity (Tameshige et al., 2013). These driver and reporter plants were crossed, and the resulting two lines #3 and #7 were analyzed (**Figures 2B–D**, **Supplementary Figure S2**).

Importantly, when grown under standard conditions around 23°C, the crossed lines did not show any VENUS fluorescence in any part of the seedlings with or without DEX (**Figure 2B**). Even when grown on DEX-containing media for six days, VENUS fluorescence was undetectable in the seedlings, indicating that the dual regulation of transgene expression fully functioned (**Figure 2B**). Upon treatment at 37°C for 10 min, VENUS fluorescence was detected only in the DEX-treated seedlings, but not in the non-treated seedlings at 24 h after HS treatment. The seedlings continuously grown on DEX-containing media showed stronger VENUS signals than those treated with DEX for 24 h. When HS treatment was performed for 60 min, both types of DEX treatments gave rise to the induction of strong VENUS fluorescence almost ubiquitously throughout the seedlings, suggesting that the induction was saturated (**Figure 2B**). Under these HS conditions, the seedlings without DEX treatment also exhibited sparse but detectable VENUS fluorescence throughout the seedlings, suggesting that strong HS stimuli overcame the control of the DEX-regulated system (**Figure 2B**). Magnified images of the leaf and root tissues of the 60 min-treated seedling indicated that VENUS fluorescence was induced in almost all cell types following adequate HS treatment (**Figures 2C, D**).

Next, we analyzed the temporal kinetics of transgene expression in the dual regulation system using lines #3 and #7 (**Supplementary Figure S2**). We first incubated the seedlings on DEX-containing MS medium for 24 h and subsequently cultured them at room temperature or 37°C for 60 min. All seedlings were then placed on the same DEX-containing MS medium and monitored using time-lapse imaging. The intensity of the VENUS signal was detected only in HS-treated seedlings at approximately 6 h and increased up to 24 h after HS. These data indicate that our newly developed p*HSP18.2v2*-mediated CRE-GR/*loxP* system accomplishes a series of molecular events: HS-stimulated CRE-GR expression, DNA recombination between *loxP* sites, and full level expression of the gene of interest, within 24 h after HS. In addition, DEX-dependent recombination was shown to be triggered only with an appropriate HS treatment, such as 37°C for 60 min, in the presence of DEX. Taken together, these data demonstrate that HS- and DEX-dependent dual regulation of transgene expression offers a strictly controllable expression system, allowing for the analysis of morphogenic or cytotoxic genes in this system.

### 3.3 Optimization of the local HS induction method for the targeted p*HSP18.2v2*-mediated CRE-GR/*loxP* system

The IR-LEGO system enables local transgene expression by eliciting HS responses at single-cell resolution in a given tissue (Kamei et al., 2009). Our p*HSP18.2v2*-mediated CRE-GR/*loxP* system may become a powerful tool when combined with IR-LEGO for the induction of transgene expression in targeted single cells. The successful application of the IR-LEGO system to *A*. *thaliana* and *Marchantia polymorpha* has been previously demonstrated (Deguchi et al., 2009; Maruyama et al., 2017; Hwang et al., 2019). However, these previous studies have not sufficiently achieved the desired HS-mediated gene induction in a given plant tissue. Owing to its highly tight transcriptional regulation, the p*HSP18.2*v2-mediated CRE-GR/*loxP* system can also serve as a good experimental system to determine the optimal conditions for CRE/*loxP* recombination in targeted plant cells with IR-LEGO. Therefore, we assessed the p*HSP18.2v2*-mediated CRE-GR/*loxP* system in combination with IR-LEGO.

**Figure 3:**
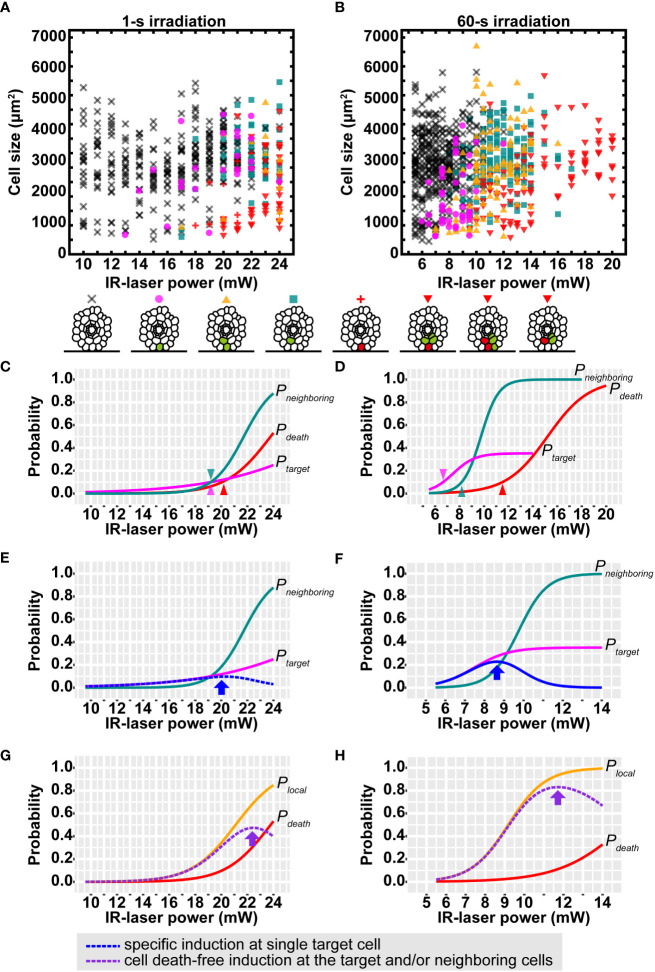
Efficiency of VENUS expression after IR-laser irradiation to epidermal cells in roots. **(A, B)** Distribution of the VENUS expression patterns after IR-laser irradiation for 1 s **(A)** and 60 s **(B)**. The cross, circle, triangle, square, plus, and inverted triangle indicate the resultant patterns of no fluorescence, VENUS fluorescence only in the irradiated cell, VENUS fluorescence in the irradiated and other cells, V VENUS fluorescence only in non-irradiated cell(s), PI fluorescence (cell death) only in the irradiated cell, and both PI and VENUS fluorescence in the irradiated and other cells, respectively. These patterns are schematically shown below the graphs, where cells with VENUS and PI fluorescence are indicated by green and red colors, respectively. **(A)** 10.0–16.0 mW (*n* = 20), 17.0–19.0 mW (*n* = 30) and 20.0–24.0 mW (*n* = 40). **(B)** 5.5–8.0 mW (*n* = 50), 8.5–11.0 mW (*n* = 40), 11.5–14.0 mW (*n* = 30) and 15.0–20.0 mW (*n* = 9). **(C, D)** Sigmoid curves determined by logistic regressions of the data in **(A, B)**. The probability of VENUS expression in the single target cell (*P_target_*) and neighboring cells (*P_neighboring_*) and cell death probability (*P_death_*) are shown as magenta, blue-green, and red lines, respectively. Arrow heads: the IR-laser power where the probabilities reach 10%. **(E, F)** The calculated probability of VENUS expression in the single target cell without that in the neighboring cells (blue dotted line). Arrows indicate the highest probabilities. Magenta and blue-green lines are the same as those in **(C, D). (G, H)** The calculated probability of VENUS expression in the target and/or the neighboring cells without cell death (purple dotted line). Arrows indicate the highest probabilities. Brown lines are the same as those in **(C, D)**. Orange lines represent the probability of VENUS expression in the target and/or the neighboring cells (*P_local_*) from logistic regressions. The IR-laser irradiation time is 1 s **(C, E, G)** and 60 s **(D, F, H)**.

The IR laser easily reached the outermost layer *in vivo* on the cover glass side. Therefore, we sought the optimal conditions of the IR-LEGO system in the epidermal cells of Arabidopsis roots growing between the DEX-containing media and cover glass (**Supplementary Figure S3**). In this system, the power and duration of the IR laser irradiation can be modified to achieve an optimal HS response in a given tissue. As for the duration, 1-s IR laser irradiation at a certain range of laser power has been widely used to induce gene expression in animal systems (Deguchi et al., 2009; Kimura et al., 2013; Kawasumi-Kita et al., 2015; Miao and Hayashi, 2015; Hasugata et al., 2018). In addition, previous studies have suggested that the 60-s irradiation for stable HS resulted in more efficient gene induction than the 1-s irradiation (Kamei et al., 2009). Since the extended IR-laser duration is easily applicable to sessile plants (Hwang et al., 2019), we examined the differences in inducibility between 1- and 60-s irradiation at various IR-laser powers in more detail. Using the above VENUS reporter lines, inducibility was assessed and categorized by microscopic observation of CRE-GR/*loxP*-mediated VENUS induction around IR-laser-irradiated cells (**Figures 3A, B**; **Supplementary Figure S4**). Here, we used propidium iodide (PI) to visualize dead cells resulting from excess IR-laser irradiation (**Supplementary Figures S4F–H**). Compared with 1 s irradiation, 60 s irradiation seemingly exhibited higher inducibility, as reported in previous studies (Kamei et al., 2009). In the 1 s irradiation, as the IR-laser power was increased, both the frequencies of VENUS induction and cell death simultaneously increased (**Figure 3A**). In contrast, the 60 s irradiation successfully induced VENUS fluorescence without causing cell death at a broader range of laser powers than the 1 s irradiation (**Figure 3B**). In addition, we observed that cell size may affect the inducibility of VENUS (**Figures 3A, B**).

Despite such a large number of irradiation attempts under various conditions, no conditions were found to achieve 100% recombination at the target cell. Nevertheless, the following trends were observed.

- Low laser power (less than 10 mW for 1 s or 5.0 mW for 60 s) induces “no recombination.”
- The probability of “recombination in the target and neighboring cells” gradually increases as the laser power increases.
- The probability of “cell death in the target cell” also increases as the laser power increases.
- High laser power (more than 24 mW for 1 s or 17.0 mW for 60 s) induces “cell death” in more than 50% cases.

Considering these points, we sought optimal conditions for the practical use of IR-LEGO in plants. The meaning of the best condition is redefined as follow1. A condition that shows the highest probability of inducing recombination in the single target cell but not in other cells.

2. A condition that shows the highest probability of inducing recombination in one or more cells among the target and nearby cells without cell death in any cell.

The former condition is important in experiments aimed at expressing a gene in only one cell from a pair of neighboring cells (e.g., between two daughter cells after division, between an epidermal cell and the underlying cortex cell, or between a trichoblast and an atrichoblast). The latter condition is useful for local manipulation of cellular gene expression regardless of single cell (e.g., manipulation of root meristem cells but not in transition-zone cells or manipulation of right-half cells but not in left-half cells). To determine such conditions, we calculated the following relevant probabilities:

*P_target_*: recombination probability at the single target cell
*P_neighboring_*: recombination probability at one or more neighboring cells of the target
*P_local_*: recombination probability at the target and/or neighboring cells
*P_death_*: cell death probability at the target and/or neighboring cells

The basic assumption here is that each probability increases at higher laser powers, resulting in a sigmoidal curve. Each response curve for the highest likelihood was estimated using standard or modified logistic regression (see Materials and methods). In both the cases of 1 and 60 s irradiation, the obtained response curves indicate that *P_target_*, *P_neighboring_* and *P_death_* increase in this order as the laser power is increased. Notably, the three raising points, at which each probability increases beyond 10% inducibility, were separated better in the case of 60 s irradiation than that of 1 s irradiation (**Figures 3C, D**). *P_local_* calculated from the regression of the data was consistent with that indirectly calculated from *P_target_* and *P_neighboring_* considering their relationship: *P_local_* ≈ *P_target_* + *P_neighboring_* – *P_target_* × *P_neighboring_*. Then, *P_target_* × (1 – *P_neighboring_*) and *P_local_* × (1 – *P_death_*) were calculated to find conditions 1 and 2. Finally, we concluded that condition 1 is met by applying 20.0 mW for 1 s (**Figure 3E**; arrow) or 8.5 mW for 60 s (**Figure 3F**; arrow), and that condition 2 is met by applying 22.5 mW for 1 s (**Figure 3G**; arrow) or 11.5 mW for 60 s (**Figure 3H**; arrow). These probability models evidently indicate that 60 s irradiation gives preferable results at higher probabilities than 1 s irradiation under both the conditions 1 and 2. Taken together, 60 s was consistently better than 1 s as the irradiation time.

The aforementioned probability models were obtained from all the data, reflecting the probabilities in a cell of average size of approximately 3,000 µm^2^ in this study. However, the induction was slightly biased toward smaller cells, as mentioned above. To gain more insight into the effect of cell size, we filtered large and small cells from the data and calculated the probabilities using the same logistic regression. The resulting curve indicated that all probabilities, *P_target_*, *P_neighboring_*, *P_local_* and *P_death_*, increased if the cell sizes were small, and vice versa (**Supplementary Figure S5**). To test whether the effects of this cell size can occur by chance, we performed likelihood-ratio tests comparing cell size-ignored and -included models. Significant effects were detected (3.12e^-16^< *p*< 2.03e^-3^) in most cases, except for *P_target_* of 1 s irradiation (*p* = 0.0531). Such size effects imply that care should be taken under the optimal conditions of irradiation time and power. If the target cell is relatively small, such as meristematic cells or large-like vacuolated cells, a lower or higher laser power, respectively, is recommended to obtain preferable induction. From the cell size-included probability models, a series of optimal laser powers can be predicted for any size of cells (**Supplementary Figure S6**).

**Figure 4:**
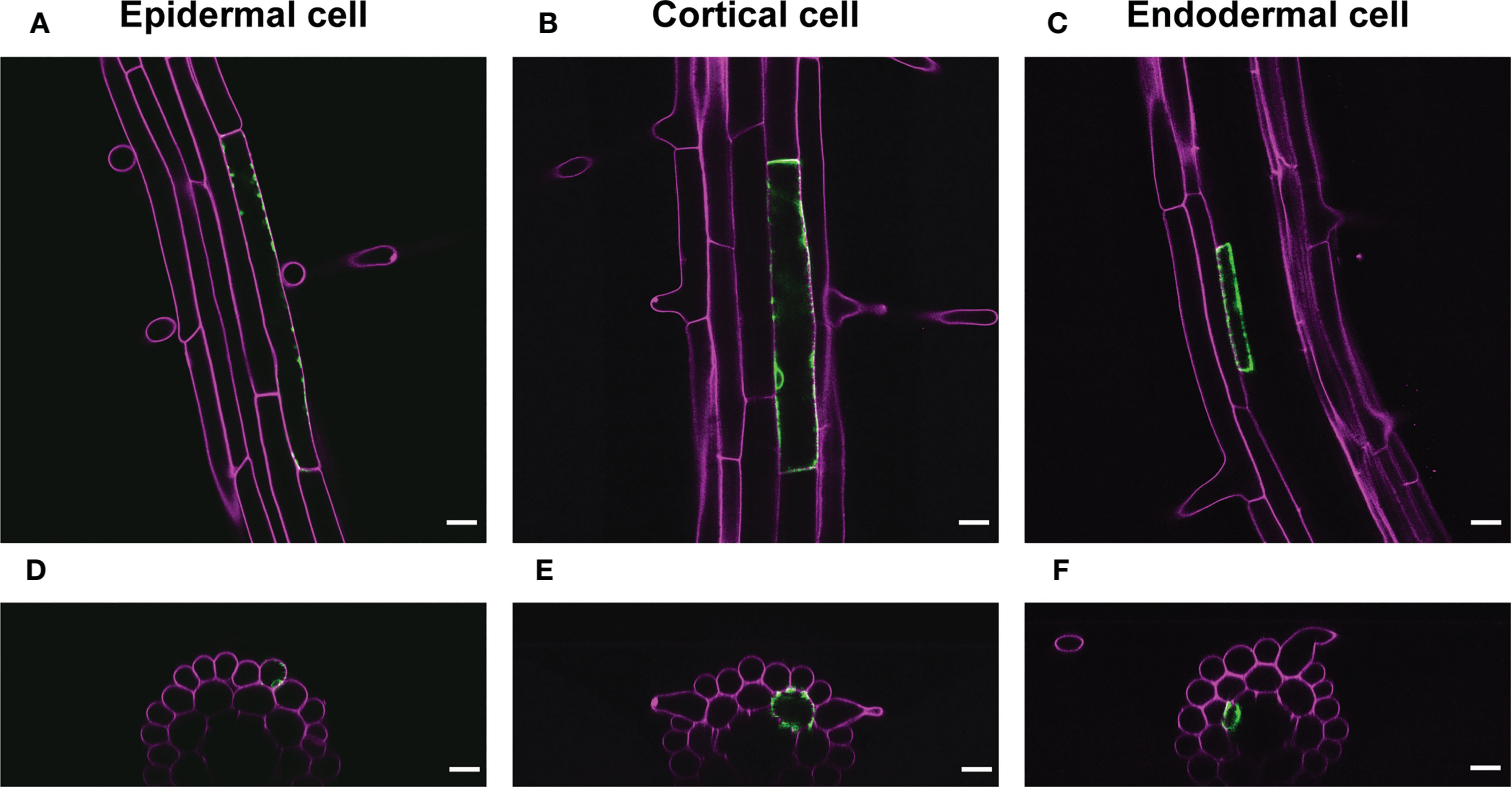
Representative images of VENUS expression at the single cell. **(A-C)** VENUS fluorescence (green) was detected only in the epidermal cell **(A)**, cortical cell **(B)**, or endodermal cells **(C)** 1 d after IR-laser irradiation. **(D–F)** The optical cross sections of the roots shown in **(A–C)**, respectively. Samples were stained with PI (magenta). Scale bars represent 20 μm. IR-laser irradiation for 60 s was carried out with 9.0-mW output to the epidermal cell (4,714 μm^2^) **(A, D)**, 10.0-mW output to the cortical cell (4,417 μm^2^) **(B, E)**, and 8.5-mW output to the endodermal cell (845 μm^2^) **(C, F)**. Three-dimensional reconstructions were performed from the confocal images (**Movies S1-3**).

We also applied a range of IR-laser outputs to cortical and endodermal cells as well as epidermal cells in roots with 60 s irradiation. The attendant VENUS fluorescence at the single cells in these tissues empirically required the output according to the cellular volume (**Figure 4**; **Movies S1-3**). These data also support our hypothesis that the thermal capacity derived from cellular size affects the efficiency of induction of gene expression through HSR.

### 3.4 Application of the targeted p*HSP18.2*v2-mediated CRE-GR/*loxP* system in leaf tissues using IR-LEGO

**Figure 5:**
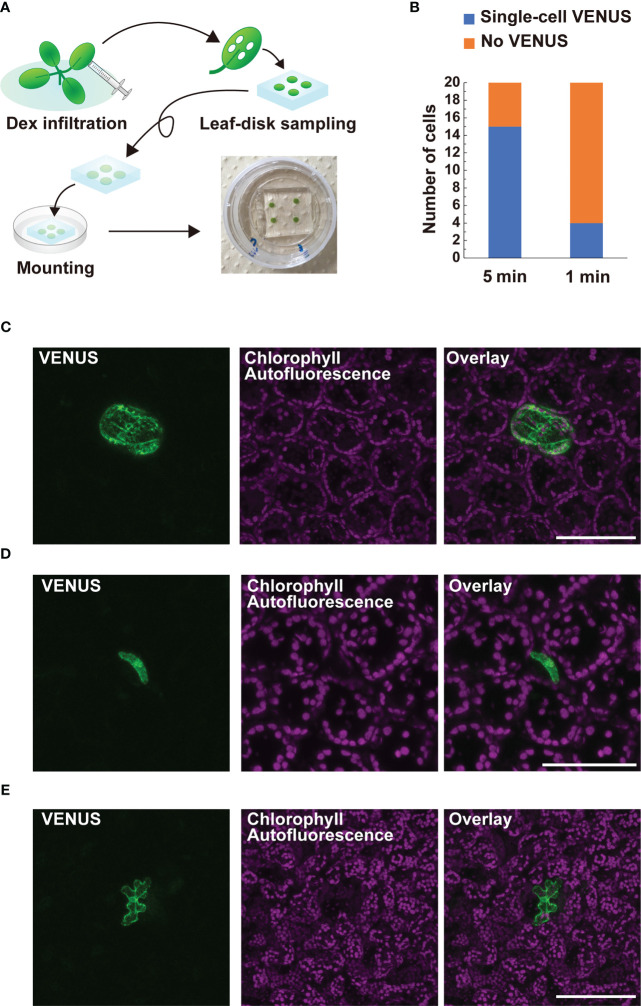
Application of targeted p*HSP18.2v2*-mediated CRE-GR/*loxP* system equipped with IR-LEGO to leaf tissues. **(A)** Schematic diagrams describing how leaf samples were prepared for IR-LEGO experiments. First, the leaves of 14-day-old plants grown on 1/2 MS media were fully infiltrated with 1/2 MS liquid media containing DEX. Leaf disks were prepared from the infiltrated leaves using a biopsy punch (diameter 3 mm) and then placed on a block of 1/2 MS media containing DEX. The leaf disk-side of the block was directly placed onto the glass part of a 35-mm glass bottom dish and visible air bubbles between the leaf disk and glass were carefully removed by pressing the media gently. **(B)** The frequencies of irradiation experiments resulting in successful single-cell VENUS induction (blue) within 20 trials targeting mesophyll cells are shown for irradiation durations of 5 and 1 min, respectively. **(C–E)** Maximal intensity projection of magnified confocal images of single-cell VENUS induction in a mesophyll cell **(C)**, a stomatal guard cell **(D)**, and an epidermal cell of the leaf tissues **(E)**. Representative images of line #3-3 **(D)** and #3-7 **(C, E)** were presented. VENUS, chlorophyll-derived autofluorescence, and the merged images are shown. Scale bars represent 100 µm **(C, E)** and 75 µm **(D)**, respectively.

CRE/*loxP* recombination using the IR-LEGO system has been reported in the roots and female gametophytes, but not in leaves, in Arabidopsis (Deguchi et al., 2009; Maruyama et al., 2017; Hwang et al., 2019). This may limit further application of the targeted *pHSP18.2v2*-mediated CRE-GR/*loxP* system assisted by IR-LEGO to genetic mosaic dissection of various biological events in the leaf tissues. Therefore, we examined whether our newly defined conditions could be applied to targeted gene induction in leaf tissues. Here, we used DEX-treated Arabidopsis leaf disks settled between gel media and glass (**Figure 5A**). When our optimized conditions for root cells were applied to leaf mesophyll cells, single-cell VENUS expression was achieved in only four out of 20 irradiation trials, suggesting possible insufficiency of the heat stimuli for those cells (**Figure 5B**). This result prompted us to examine a longer irradiation time with the same laser power in the same experimental setup, as the results with roots emphasized the effect of elongated irradiation time rather than that of higher laser power (**Figures 4A, B**). As expected, 5 min irradiation dramatically increased the induction rate of leaf mesophyll cells (**Figure 5B**). Using the same conditions, we also succeeded in detecting the VENUS signal in single stomatal guard cells and epidermal cells, although the efficiency of the recombination in these cells was not as high as that in mesophyll cells (**Figures 5C, D**). These data demonstrate that our system, in combination with IR-LEGO, can be successfully applied to activate CRE-GR/*loxP* recombination in various cell types of leaf tissues with minimum optimization, in addition to the root system.

## 4 Discussion

In this study, we present a newly developed system that enables the expression of a gene of interest in targeted single cells. There exist a number of inducible and conditional gene expression systems (Corrado and Karali, 2009). Most of them depend on the use of chemicals and are unsuitable for achieving gene expression in a single cell or in a small area consisting of several cells of the whole body owing to technical difficulties in the fine-tuned application of chemicals. Together with such chemical-based conditional expression systems, the use of promoters active only in particular cell types enables more cell type-specific gene expression, but the selection of promoters limits the range of the cell types to express a gene of interest. Optogenetics, which is mainly established using plant-based protein domains, appears to have intrinsic limitations in their application to plants (Christie and Zurbriggen, 2021). Therefore, IR-LEGO can be used as an alternative optogenetic method that enables targeted single-cell mosaic analysis in plant fields.

As presented in this study, targeted conditional gene expression in a desired cell or a desired part of a tissue in a body would uncover a new avenue to study cell-to-cell communication, such as genetic chimera-based studies. Chimeric and mosaic analyses in plants and animals, prior to the era of molecular biology, have been unique approaches for dissecting how multicellular organisms coordinated their growth and development at the cellular level (McLaren, 1976; Tilney-Bassett, 1991). Grafting has also been used to study systemic signaling in plants (Tsutsui and Notaguchi, 2017). The emergence of transgenic approaches based on inducible gene expression systems in restricted cell(s), such as the HS-mediated CRE/*loxP* system, has further promoted the genetic understanding of the molecular basis of organism growth and development (Frank and Chitwood, 2016).

The conventional p*HSP18.2* has been widely and successfully used to express genes of interest in an HS-dependent manner in plants, including Arabidopsis; however, its basal transactivity in the vasculature and other tissues of Arabidopsis plants without HS has been recognized, as is the case for other *HSP* genes (Takahashi et al., 1992; Yabe et al., 1994; Crone et al., 2001; Deguchi et al., 2009). Indeed, gene expression databases show that transcripts of *HSP18.2* and other *HSPs* are detected in specific types of cells such as root endodermis and guard cells without HS treatment (Winter et al., 2007; Zhang et al., 2021). This may interfere with attempts to express stress-causing, cytotoxic, or morphogenic genes that affect developmental processes under the control of those *HSP* promoters. We assessed two promoters: p*HSP18.2* and p*4xHSE* in order to establish a promoter showing high inducibility without basal transactivity. The HS response is mediated by a group of HSFs that bind to HSEs in the regulatory sequences of target genes. Among the variations in HSE sequences, an idealized HSE sequence, 5′-AGAACGTTCTAGAAC-3′, was obtained from the human *HSP70.1* promoter (Cunniff and Morgan, 1993). The use of eight tandem repeats of this HSE sequence, together with the CMV minimal promoter, successfully achieved heat-shock-specific induction of a gene in medaka fish (Bajoghli et al., 2004). However, our synthetic promoter consisting of four tandem repeats of the identical HSE fused to the minimal p*35S* promoter (p*4xHSE*) did not exhibit HS-specific strong inducibility of the reporter gene in Arabidopsis (**Supplementary Figure S1**). The repeat number, the HSE sequence used, and/or other parts of our promoter design did not fit Arabidopsis. A synthetic promoter constructed by combining the *cor15A* promoter and a soybean-derived HSE sequence has been reported to exhibit HS-specific activity (Maruyama et al., 2017). A study of the promoter of a small HSP, *sHSP26*, points to the functional significance of the 5′-UTR in strong HS-triggered expression (Khurana et al., 2013). These examples suggest the importance of plant-derived HSE sequences and the intrinsic 5′-UTR.

The Arabidopsis intrinsic promoter, *pHSP18.2v2*, was successful in terms of no detectable basal activity and high HS-dependent induction in all the cell types examined, presumably due to the presence of putative regulatory *cis* element(s) lying at the newly added 149-bp promoter sequences. However, preferentially stronger HS-mediated activity in particular cell types, such as vasculatures and stomatal guard cells (**Figures 1F, G**), was still observed, implying that HS sensitivity depends on cell type. A recent study on single-cell transcriptomes showed that some root cells and stomatal guard cells show similar transcriptional profiles and appear to be hypersensitive to environmental conditions (Zhang et al., 2021). The regulatory mechanisms of HS-responsive gene expression have been demonstrated to rely on molecular events regulated by HSFA2 binding, histone modification, and JMJ domain proteins (Lämke et al., 2016; Yamaguchi et al., 2021). Further studies dissecting HS-mediated gene expression, especially the molecular basis governing the cell-type specificity of HS-mediated gene expression, should lead to the design of more efficient HS-specific and versatile promoter sequences.

Our data demonstrated that the double regulation of the CRE/*loxP* system by p*HSP18.2v2* as well as the steroid hormone assures reliable HS- and DEX-specific expression of a gene of interest in Arabidopsis. Although, under moderate HS conditions, the p*HSP18.2v2*-mediated CRE-GR/*loxP* system itself can be used to perform mosaic analyses owing to the stochastic nature of CRE-mediated recombination, a combination of the system and IR-LEGO enabled HS-mediated gene expression in a targeted manner. Because IR-LEGO is based on IR laser-mediated “heating” of the irradiated cells, physical and chemical factors, such as the cellular volume of a given biological system, strongly affect thermal efficiency; that is, the rate of successful HS-mediated gene expression in the targeted cells. This implies that once suitable parameters are adjusted, IR-LEGO can become a solid tool in a given biological system. Our data also support the idea that adjusting the IR laser power to a target cell size increases the efficiency of gene induction (**Figures 3A, B**). In this study, we screened a range of IR laser powers at two different irradiation durations for the optimal conditions to achieve single-cell gene expression using IR-LEGO with the p*HSP18.2v2*-mediated CRE-GR/*loxP* system in Arabidopsis roots (**Figures 3A, B**). To the best of our knowledge, we did not find a condition that resulted in 100% efficiency in the target cell. To overcome the wide range of variations in the obtained dataset, possibly due to the stochastic nature of the CRE recombination events and the cell size variations, we took advantage of statistics-based mathematical modeling to describe an optimal condition from the experimental data. In this analysis, the cell volumes were approximated to the longitudinal cell area, considering the typical characteristics of the longitudinal elongation of root cells. Our calculation assumption suggested an optimal IR laser power using 60 s irradiation at a particular cell size of approximately 3,000 µm^2^, which is the average cell size measured in this study (**Figures 3C–H**). Using this optimal condition, we successfully expressed VENUS in the targeted single cells of the cortical, endodermal, and epidermal layers of Arabidopsis roots (**Figure 4**). Our finding that the optimal IR laser power depends on the cell size and that the detailed probability can be predicted from our model (**Supplementary Figure S6**) will provide further useful reference information in various experimental situations.

Further efforts to increase the efficiency will make the system more useful. The kinetics of DNA recombination reactions catalyzed by CRE have been well studied. The CRE protein dimer and a DNA double strand of *loxP* site form a complex, and the two dimers of CRE form a tetramer complex together with the two DNA double strands. This complex allosterically activates CRE, leading to DNA strand breaks and reunion reactions (Duyne, 2001). The complex formation rate increases in response to CRE protein concentration; thus, a high expression level of CRE can increase efficiency. Recently, some plant-derived 3′-regulatory sequences or terminators have been reported as useful modules for higher level expression of transgene (de Felippes et al., 2022). The rate of DNA strand break and reunion reactions might be difficult to increase by protein engineering because no hyperactive mutation has been known for CRE. However, other classes of DNA recombinases appear to be potential candidates for more efficient recombination. Site-specific recombinases other than CRE have been found in bacteria and yeast (e.g. FLP, Dre, and Vika), and their applicability has been validated in mammals (Karimova et al., 2018). The duration of CRE expression also has an impact on reaction efficiency (Saidi et al., 2009). We observed that whole plant HS for 60 min efficiently induced CRE/*loxP* recombination in almost all cells (**Figures 2B–D**). Thus, a longer duration and/or multiple rounds of laser irradiation might lead to more efficient recombination, although such a laborious experimental operation might hamper data collection. Using mathematical modeling, we found a good condition to realize almost 100% recombination in at least one of the targeted and neighboring cells. However, cell death occurred at a certain frequency with such a high laser power. A more efficient vector system and irradiation protocol will reduce not only cell death but also damage to cellular activity.

In this study, we extended the application of IR-LEGO to Arabidopsis leaf tissue and successfully expressed VENUS in targeted single mesophyll, epidermal, and stomatal cells. In leaf tissues, longer irradiation with the same laser power as that used in root tissues resulted in a higher success rate of HS-mediated VENUS expression in the targeted single cells. This suggests that the leaf tissues have different thermal efficiency or HS sensitivity from the root tissue, possibly due to the composition of the cell wall, cellular physiological state, and/or cellular architecture. Alternatively, this may simply reflect technical issues. In this study, we mounted leaf disks between the cover slip and MS media, similar to the experimental setup used for the root tissues (**Figure 5A**). In leaf tissues, trichomes appear to create a water space between the epidermal surface and cover slip. In addition, the tissue itself is waving, which causes irregular gap spaces between the leaf surface and cover glass. Although we used a small leaf disk to minimize the effect of leaf shape, it may further emphasize the effect of trichomes generating a thick water layer (several trichomes are usually found in a leaf disk with a diameter of 3 mm). This water layer may absorb the IR laser, thereby reducing the laser output reaching leaf cells. Nevertheless, our study demonstrated that the p*HSP18.2v2*-mediated CRE-GR/*loxP* system with IR-LEGO can also be used for targeted single-cell gene expression in stomatal guard cells, mesophyll cells, and epidermal cells in Arabidopsis leaf tissue (**Figures 5C–E**). Further detailed studies on the use of IR-LEGO in leaf tissue, as well as for HS detection mechanisms in plants, would provide a better understanding and experimental setup. In this context, IR-LEGO may be useful for unveiling HS detection mechanisms at the cellular level.

Here, we present a newly developed system that enables the expression of a gene of interest in targeted single cells. Targeted conditional gene expression in a desired cell or a desired part of a tissue in the plant body, as presented in this study, would open up a new avenue for the study of gene function in broad aspects of plant biology. Tight regulation of the system will benefit those who handle genes with cytotoxic or strong morphogenic activity. The optimal conditions for the use of IR-LEGO in Arabidopsis roots, together with the newly developed p*HSP18.2v2*-mediated CRE-GR/*loxP* system, will help those who intend to express a gene of interest in the targeted single cells or the targeted part of a tissue in plants.

## Data availability statement

The original contributions presented in the study are included in the article/**Supplementary Material**. Further inquiries can be directed to the corresponding authors.

## Author contributions

TTo and TTa contributed equally to this study. SB designed the study. YK and SB directed and administered the study. TTo, SB, and EB performed IR-LEGO irradiation experiments and observations. TTa and SB generated transgenic lines. TTa designed and performed the statistical modeling. TTo, TTa, and SB wrote the original draft and JS, KT, and YK revised the final manuscript. All authors contributed to the article and approved the submitted version.
