# Supplementary material for: Targeted single-cell gene induction by optimizing the dually regulated CRE/loxP system by a newly defined heat-shock promoter and the steroid hormone in Arabidopsis thaliana
Source: Front Plant Sci. 2023 Jun 5;14:1171531. doi: 10.3389/fpls.2023.1171531 (PMC10283073; doi:10.3389/fpls.2023.1171531)
Supplement: Supplementary file 1 [file DataSheet_1.pdf]

frontiers

Supplementary Material

Targeted single-cell gene induction by optimizing the dually regulated CRE/*loxP* system by a newly defined heat-shock promoter and the steroid hormone in *Arabidopsis thaliana*

Takumi Tomoi[1,2,3†], Toshiaki Tameshige[4,5†], Eriko Betsuyaku[6], Saki Hamada[7], Joe Sakamoto[3,8], Naoyuki Uchida[9,10], Keiko U Torii[10,11], Kentaro K Shimizu[4,12], Yosuke Tamada[2,13,14], Hiroko Urawa[15,16], Kiyotaka Okada[16,17], Hiroo Fukuda[7,18], Kiyoshi Tatematsu[16,19], Yasuhiro Kamei[3,14,19,20*], Shigeyuki Betsuyaku[6*]

*** Correspondence:** Shigeyuki Betsuyaku
betsu@agr.ryukoku.ac.jp
Yasuhiro Kamei
ykamei@nibb.ac.jp

Figure S1

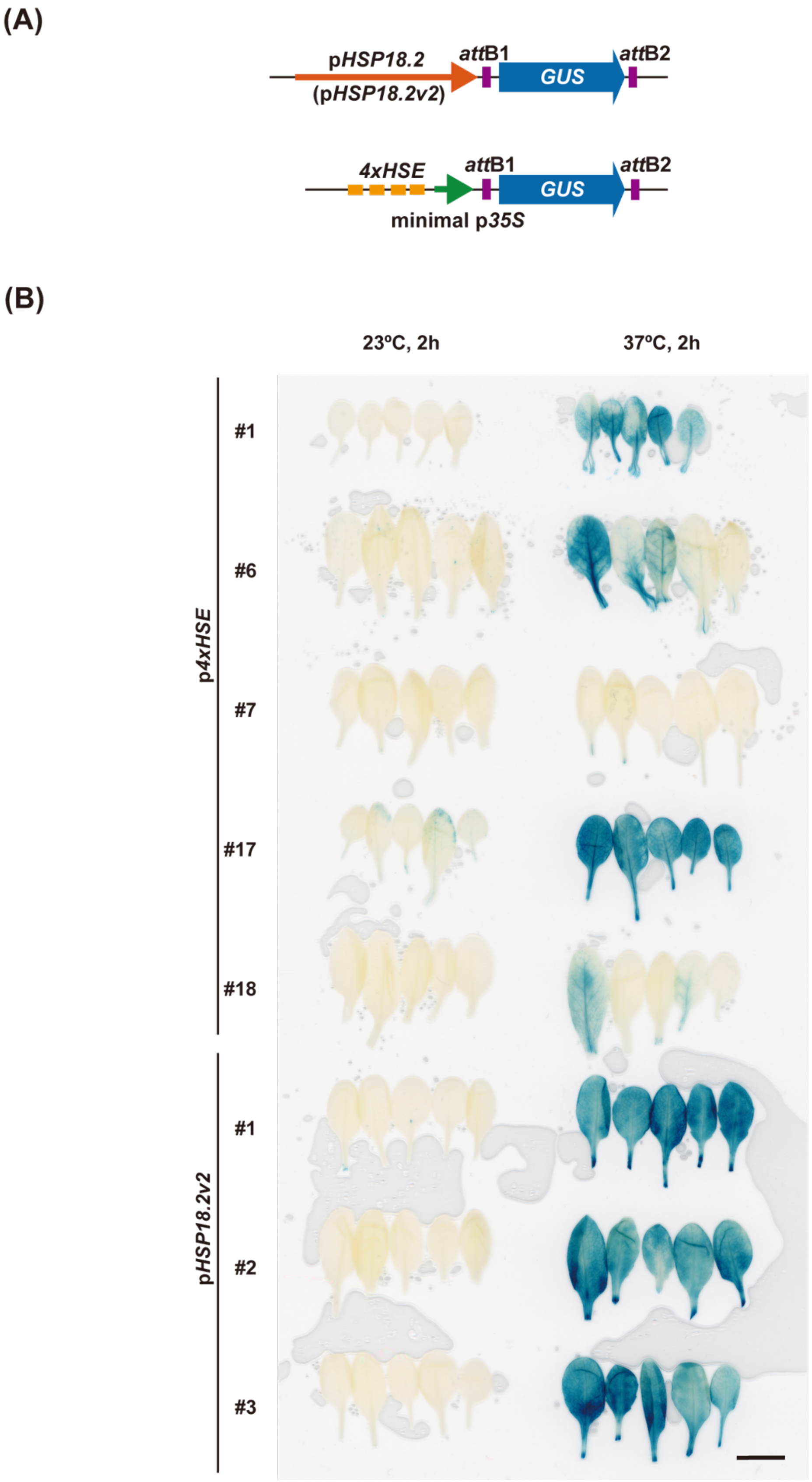
(A)
pHSP18.2
(pHSP18.2v2)
attB1
GUS
attB2
4xHSE
attB1
GUS
attB2
minimal p35S
(B)
23ºC, 2h
37ºC, 2h
p4xHSE
#1
#6
#7
#17
#18
pHSP18.2v2
#1
#2
#3

Supplementary Figure 1. Comparison of GUS reporter activity between p*HSP18.2v2*:*GUS* and p*4xHSE*:*GUS* reporter plants. **(A)** Schematic drawing of the reporter constructs used in this study. Either p*HSP18.2v2* or p*4xHSE* containing four tandem repeats of HSE fused to the minimal p*35S* promoter sequences were cloned into the pAM-PAT-GW vector, and the *GUS* reporter gene was introduced downstream of the promoters. **(B)** Images of GUS-stained p*4xHSE*::*GUS* and p*HSP18.2v2*:*GUS* reporter leaves. Five leaves derived from five different plants of a reporter line (five and three homozygous lines for p*4xHSE:GUS* and p*HSP18.2v2:GUS*, respectively) were treated at either 23 °C or 37 °C for 2 h. GUS staining of leaves was performed at 37 °C overnight. Scale bar represents 1 cm.

Figure S2

(A)

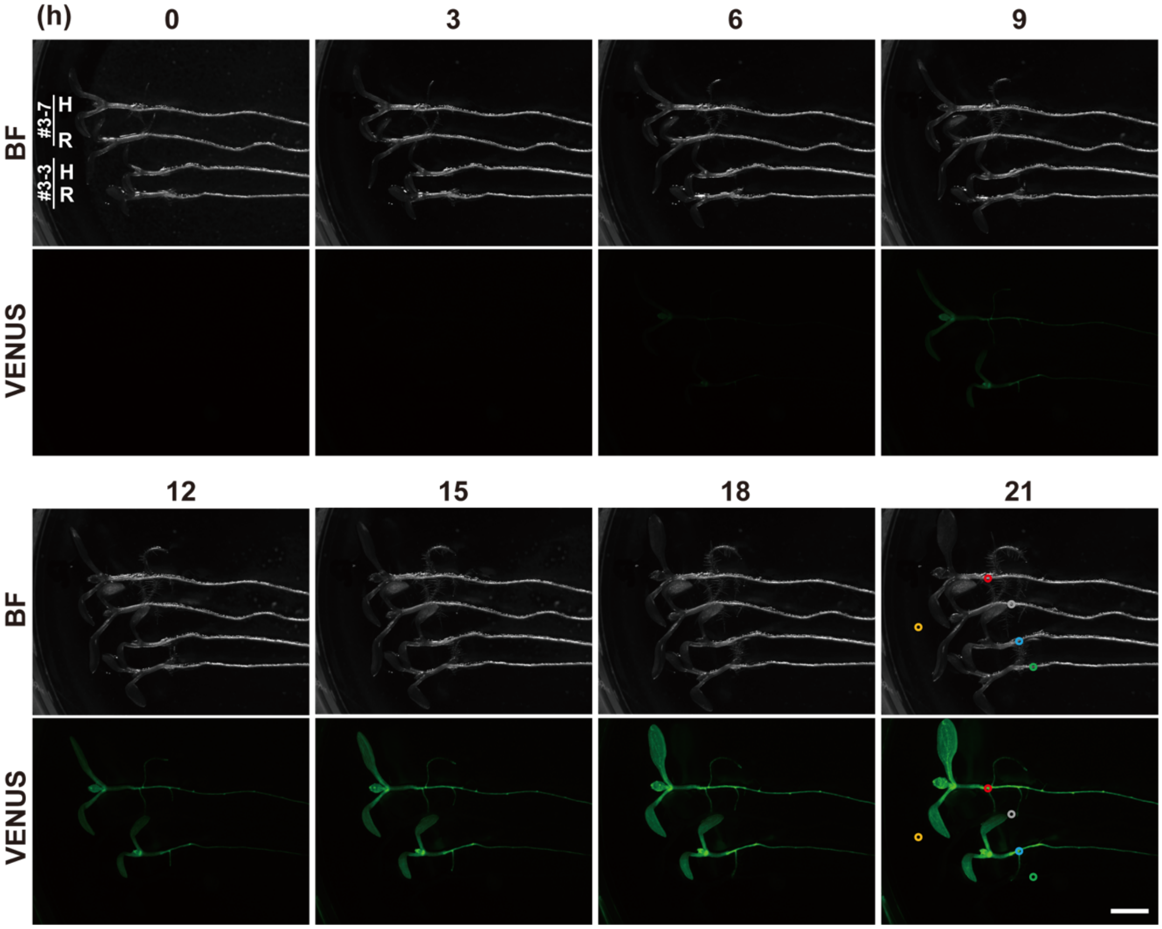
(h)
0
3
6
9
BF
#3-7
H
R
#3-3
H
R
VENUS
12
15
18
21
BF
VENUS

(B)

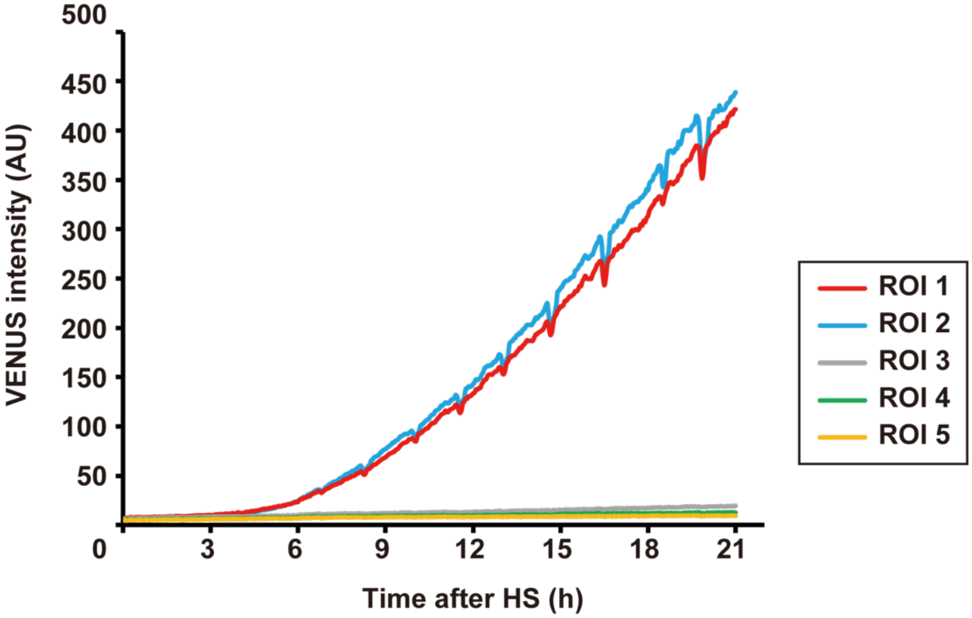
VENUS intensity (AU)
500
450
400
350
300
250
200
150
100
50
0
3
6
9
12
15
18
21
Time after HS (h)
ROI 1
ROI 2
ROI 3
ROI 4
ROI 5

Supplementary Figure 2 Temporal kinetics of VENUS induction upon HS treatment. **(A)** Images of the transgenic seedlings containing both the driver and reporter constructs upon various treatments. Two seedlings of two lines (Lines #3-3 and #3-7) grown on 1/2MS media containing DEX were treated either at 23 °C (R) or at 37 °C (H) for 60 min, respectively. Bright field (BF) and VENUS images at the indicated time points (h) after the treatments were presented. **(B)** VENUS intensity profiles of the five regions-of-interests (ROIs). Five ROIs are shown as colored circles in the BF and VENUS images at 21 h in **(A)**, and the same colors are used in the profiles. ROI 5 shown as an orange circle was used as a background. Scale bar represents 2.5 mm

Figure S3

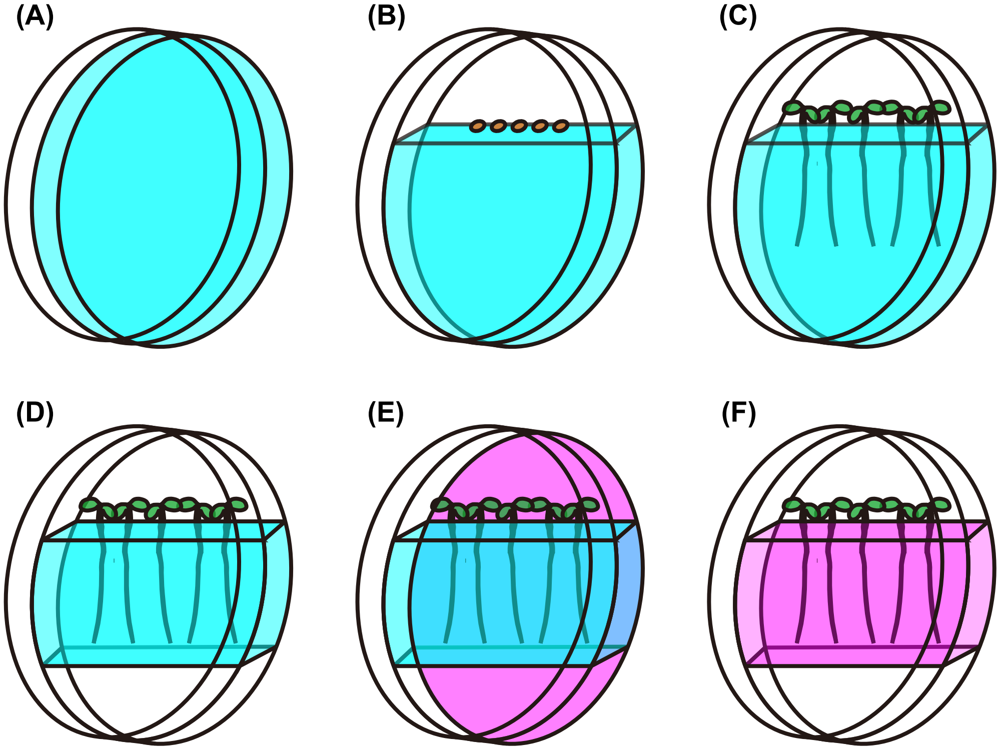

Supplementary Figure 3. Schematic images of the procedure used to make root samples for IR-laser irradiation. **(A)** The medium in glass bottom dishes. **(B)** Part of the medium was removed, and seeds were placed onto the line between the glass and cut surface. **(C)** Three-day-old seedlings after 3-d stratification. **(D)** The medium below the root tips was removed. **(E)** PI solution was added to the upper and lower parts of the sample. **(F)** Excess PI solution was removed and washed with distilled water.

Figure S4

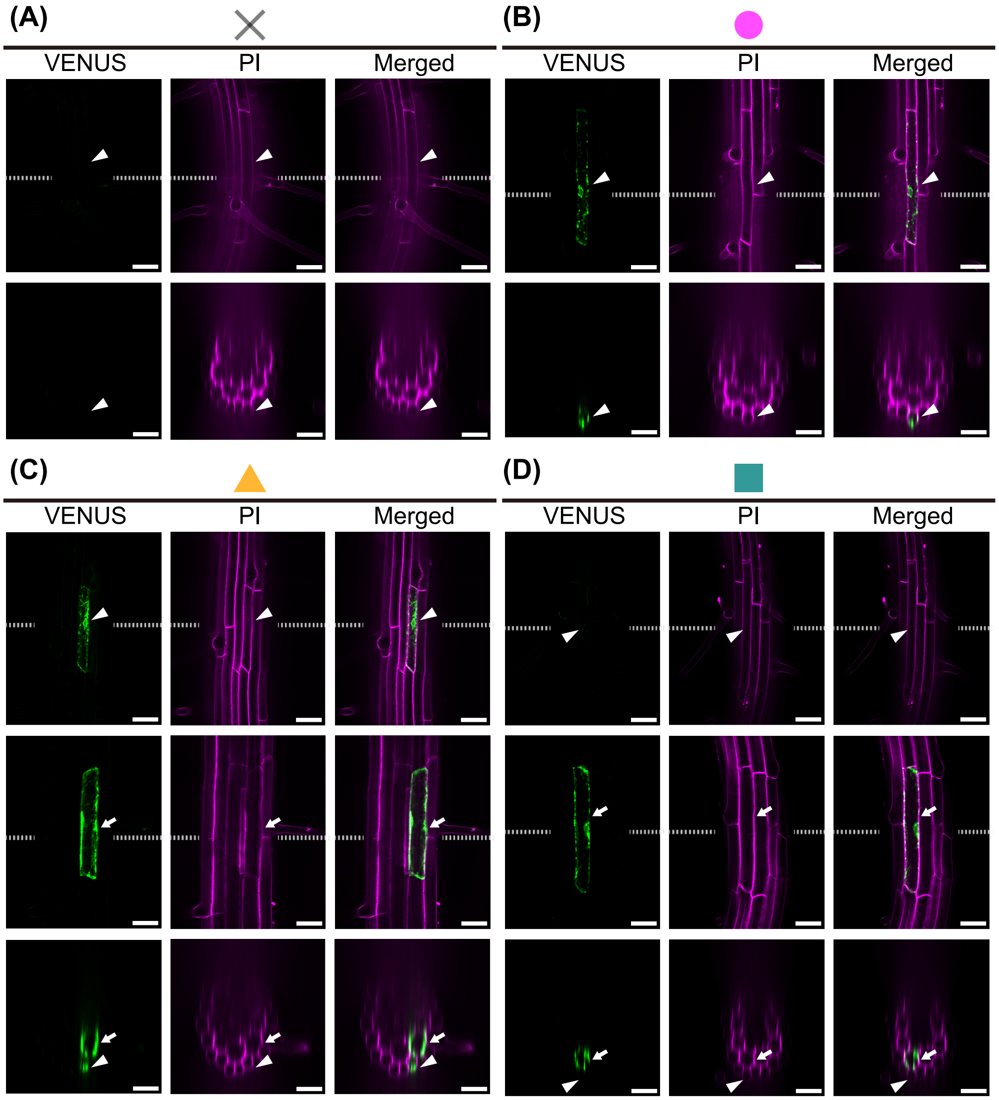

(A)
VENUS
PI
Merged
(B)
VENUS
PI
Merged
(C)
VENUS
PI
Merged
(D)
VENUS
PI
Merged

Figure S4

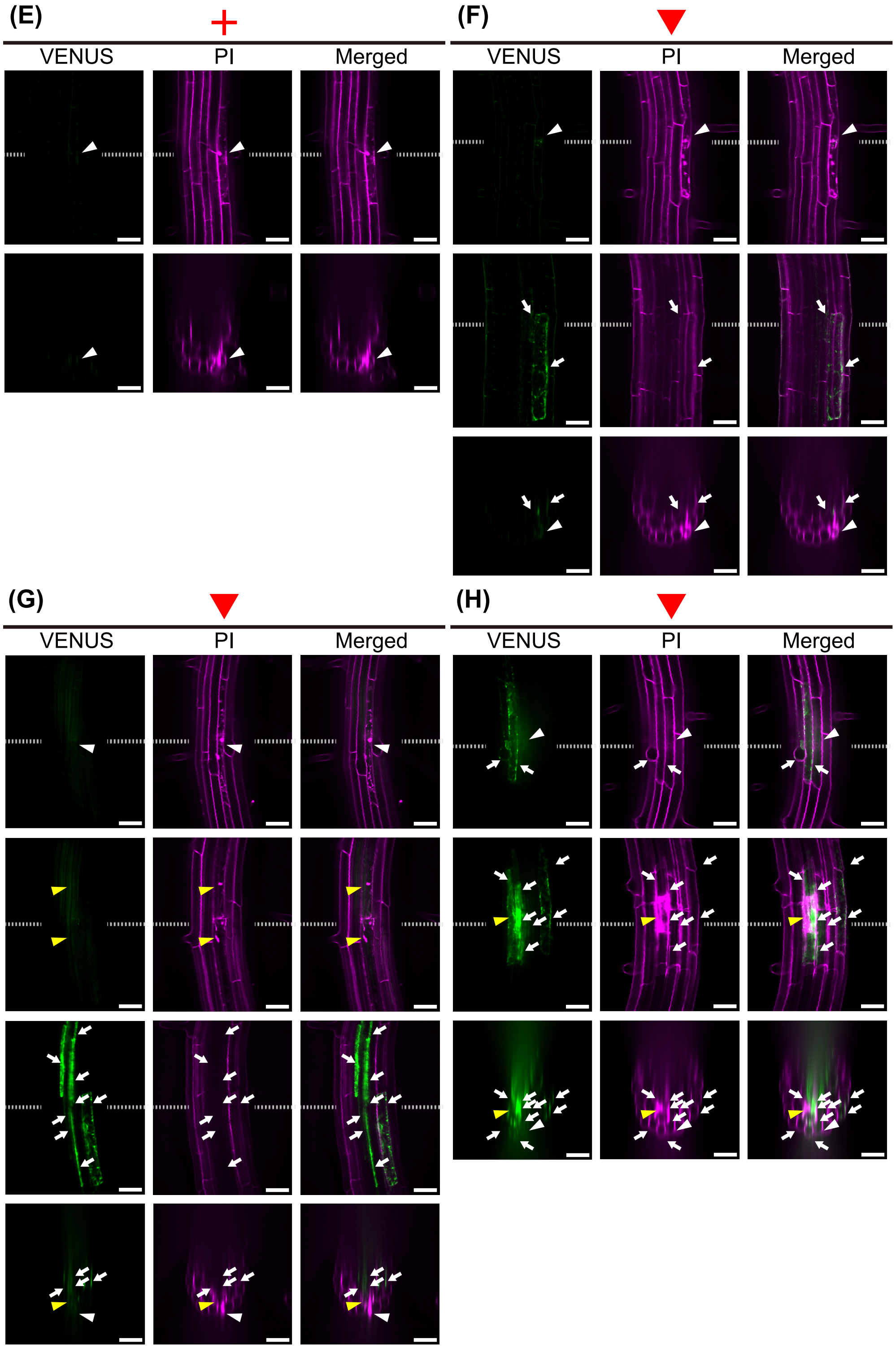
(E)
VENUS
PI
Merged
(F)
VENUS
PI
Merged
(G)
VENUS
PI
Merged
(H)
VENUS
PI
Merged

Supplementary Figure 4. Expression patterns of VENUS after IR-laser irradiation. **(A)** No fluorescence of VENUS. **(B)** VENUS fluorescence only in the irradiated cell. **(C)** VENUS fluorescence in the irradiated cell and other cells. **(D)** VENUS fluorescence only in non-irradiated cell(s). **(E)** PI fluorescence (cell death) in the irradiated cells without VENUS fluorescence. **(F)** PI fluorescence (cell death) in the irradiated cells with VENUS fluorescence in the other cells. **(G)** PI fluorescence (cell death) in the irradiated cells and VENUS fluorescence in the other cells. **(H)** PI fluorescence (cell death) and VENUS fluorescence only in non-irradiated cell(s). Dead cells were determined using the PI signal in the cells. The dotted lines correspond to the positions of the optical cross section shown below. White arrowheads, yellow arrowheads, and white arrows indicate the irradiated cells, non-irradiated cells with PI fluorescence (cell death), and non-irradiated cells with VENUS fluorescence, respectively. The meaning of cross, circle, triangle, square, plus, and inverted triangle above each panel is the same as in Figure 3. Scale bars represent 20 μm.

Figure S5

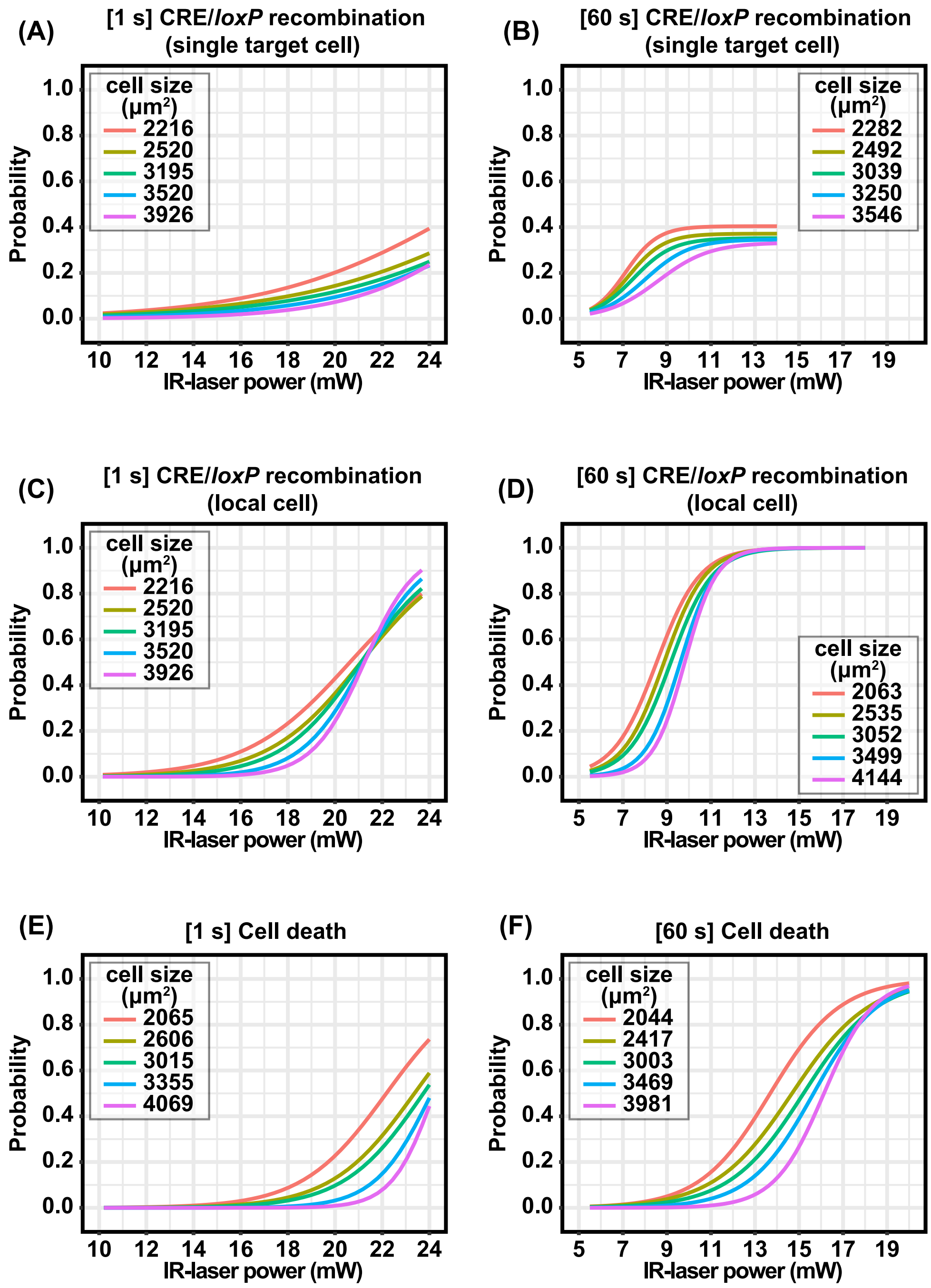
(A)
[1 s] CRE/loxP recombination
(single target cell)
cell size
(μm²)
2216
2520
3195
3520
3926
Probability
1.0
0.8
0.6
0.4
0.2
0.0
10 12 14 16 18 20 22 24
IR-laser power (mW)
(B)
[60 s] CRE/loxP recombination
(single target cell)
cell size
(μm²)
2282
2492
3039
3250
3546
Probability
5 7 9 11 13 15 17 19
IR-laser power (mW)
(C)
[1 s] CRE/loxP recombination
(local cell)
cell size
(μm²)
2216
2520
3195
3520
3926
Probability
10 12 14 16 18 20 22 24
IR-laser power (mW)
(D)
[60 s] CRE/loxP recombination
(local cell)
cell size
(μm²)
2063
2535
3052
3499
4144
Probability
5 7 9 11 13 15 17 19
IR-laser power (mW)
(E)
[1 s] Cell death
cell size
(μm²)
2065
2606
3015
3355
4069
Probability
10 12 14 16 18 20 22 24
IR-laser power (mW)
(F)
[60 s] Cell death
cell size
(μm²)
2044
2417
3003
3469
3981
Probability
5 7 9 11 13 15 17 19
IR-laser power (mW)

Supplementary Figure 5. Effect of cell size on the CRE/*loxP* recombination and cell death upon IR-laser irradiation. A series of probability curves from subset data with different cell-size filterings are shown. The irradiation duration of the IR-laser was 1 s **(A, C, E)** or 60 s **(B, D, F).** **(A, B)** CRE/*loxP* recombination at the single target cell. **(C, D)** CRE/*loxP* recombination at the single target and/or the neighboring cells. **(E, F)** Cell death at the single target and/or the neighboring cells. The cell sizes indicate the mean cell sizes in μm^2 of the corresponding subset data.

Figure S6

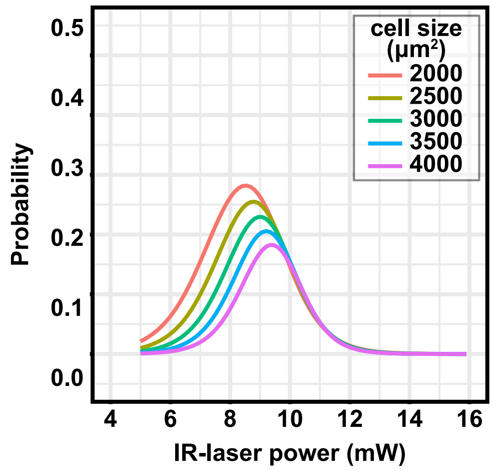

Supplementary Figure 6. The estimated optimal laser powers at different cell sizes. The Y-axis indicates the probabilities of recombination occurrence at the targeted cell without recombination at neighboring cells. Each of the recombination probabilities at the targeted (P_{target}) and neighboring cells ($P_{neighboring}$) was estimated using the logistic regression model including cell size and the interaction between cell size and IR-laser power as the explanatory variables; then, $P_{target} \times (1-P_{neighboring})$ was calculated, giving rise to the curves shown.
